# Combined Exsolution and Electrodeposition Strategy for Enhancing Electrocatalytic Activity of Ti‐Based Perovskite Oxides in Oxygen and Hydrogen Evolution Reactions

**DOI:** 10.1002/advs.202410535

**Published:** 2024-12-20

**Authors:** Shangshang Zuo, Chenchen Wang, Zhi Xia, Jiaxin Ding, Aaron B. Naden, John T. S. Irvine

**Affiliations:** ^1^ School of Chemistry University of St Andrews St Andrews Fife KY16 9ST UK

**Keywords:** bifunctional electrocatalysts, exsolution and electrodeposition, OER and HER, the double E strategy, Ti‐based perovskite oxides

## Abstract

The significant interest in perovskite oxides stems from their compositional and structural flexibility, particularly in the field of electrochemistry. In this study, the double E strategy (exsolution and electrodeposition strategies) is successfully devised for synthesizing perovskite‐based bifunctional electrocatalysts, enabling simultaneous OER and HER applications with exceptional catalytic performance. The synthesized R‐LCTFe/Ni catalyst exhibits outstanding electrocatalytic activity, delivering low overpotentials of 349 and 309 mV at 10 mA cm^−2^ for OER and HER, respectively, indicating substantial improvements in the inherent electrocatalytic activity. Moreover, the impressive stability of R‐LCTFe/Ni under alkaline conditions underscores its potential for practical water electrolysis applications. The superior bifunctional electrocatalytic performance can be attributed to the reduced charge transfer resistance and the synergistic cooperation between exsolved Fe nanoparticles and electrodeposited Ni compounds. The successful development of the R‐LCTFe/Co catalyst further confirms the transferability of the double E strategy. Compared to R‐LCTFe/Ni, the overpotential of R‐LCTFe/Co is 58 mV higher for OER, yet 48 mV lower for HER at a current density of 10 mA cm^−2^. This study provides an efficient and promising approach for the fabrication of highly active perovskite‐based electrocatalysts, contributing valuable insights into the design of bifunctional electrocatalysts for OER and HER.

## Graphical abstract

This work presents a double E strategy (exsolution and electrodeposition) to develop R‐LCTFe/Ni and R‐LCTFe/Co perovskite‐based catalysts with high bifunctional efficiency for OER and HER. The R‐LCTFe/Ni catalyst shows low overpotentials and exceptional stability, attributed to reduced charge transfer resistance and synergy between exsolved Fe and electrodeposited Ni.

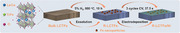

## 1 Introduction

As a strategy for addressing the challenges of energy scarcity and environmental degradation, it is crucial to prioritize the development of renewable and sustainable energy sources that can effectively replace fossil fuels in the future.^[^ ^1^, ^2^, ^3^ ^]^ Hydrogen has great potential as a zero‐carbon energy carrier that is clean and renewable, because it does not cause pollution and it is widely available.^[^ ^4^, ^5^ ^]^ One of the most efficient and environmentally friendly ways to produce hydrogen is electrochemical water splitting, which can use renewable energy sources such as solar and wind to power the process.^[^ ^6^, ^7^, ^8^ ^]^ However, the intrinsically sluggish kinetics of the oxygen evolution reaction (OER) and hydrogen evolution reaction (HER) remain a challenge in the electrochemical water splitting process.^[^ ^9^, ^10^, ^11^ ^]^ Consequently, considerable endeavors have been devoted to the development of highly efficient electrocatalysts capable of reducing energy barriers. While noble metals such as Ir/Ru oxides and Pt are widely recognized as state‐of‐the‐art electrocatalysts for OER and HER, respectively, the high cost and limited availability severely constrain their large‐scale applications.^[^ ^10^, ^11^, ^12^ ^]^ Therefore, it is crucial to design and synthesize electrocatalysts that are both economical and abundant. Perovskite oxides have emerged as a promising class of materials due to their cost‐effectiveness, compositional flexibility, tunable electronic structures, and outstanding stability.^[^ ^13^ ^]^ Although titanium‐based perovskites have been extensively utilized in solid oxide fuel cells (SOFCs), solid oxide electrolysis cells (SOECs), and photocatalysis, their potential as a bifunctional electrocatalyst for low‐temperature water splitting remains largely untapped.^[^ ^14^, ^15^, ^16^, ^17^, ^18^, ^19^, ^20^ ^]^ This may be due to the inherently poor catalytic activity of titanium‐based perovskites at room temperature. For example, Ikuya et al. systematically investigated 25 kinds of perovskite oxides for their OER catalytic performance, and found that the titanium‐based perovskites could not even reach 0.05 mA cm^−2^ at 1.8 V versus RHE.^[^ ^21^ ^]^ In recent years, our group has successfully enhanced the OER catalytic activity of titanium‐based perovskites through the redox‐exsolution method. For instance, in 2020, we improved the OER performance of CaTiO_3_ by Ni exsolution,^[^ ^22^ ^]^ and last year, we systematically investigated the influence of Co doping levels and redox conditions on the OER catalytic activity of LCTCo.^[^ ^23^ ^]^ However, their intrinsic performance still falls short of the benchmark OER catalysts, RuO_2_ and IrO_2_.

Herein, to further enhance the catalytic activity of titanium‐based perovskites, we propose a double E strategy, consisting of exsolution and electrodeposition in series, to greatly enhance the electrocatalytic activity of Ti‐based perovskite oxide La_0.25_Ca_0.65_Ti_0.95_Fe_0.05_O_3_ (LCTFe) for both OER and HER. Iron nanoparticles can be exsolved and anchored on the parent perovskite surface through reduction under a reducing atmosphere, generating a sample of R‐LCTFe. This method can increase the number of active sites and improve the performance of the perovskite. Moreover, a significant enhancement in water splitting performance can be achieved by further depositing trace amounts of nickel onto the surface of R‐LCTFe using a fast electrodeposition technique (R‐LCTFe/Ni). The synergistic effect between the iron nanoparticles and the electrodeposited nickel not only enhances electron transfer efficiency but also creates a combined catalytic performance that exceeds the sum of their individual contributions, demonstrating a 1 + 1 > 2 effect. To validate the extensive applicability of the double E strategy, we further investigate the utilization of cobalt instead of nickel during the electrodeposition process. Under the conditions of a 10 mA cm^−2^ current density, R‐LCTFe/Co, in contrast to R‐LCTFe/Ni, shows a 58 mV overpotential increase for OER and a 48 mV decrease for HER. In both R‐LCTFe/Ni and R‐LCTFe/Co, the content of active Fe and Ni (or Co) is quite small. Therefore, the double E strategy not only enhances the catalytic activity of Ti‐based perovskites but also improves the atom utilization efficiency of the active elements, resulting in increased mass activity. Consequently, the double E strategy presents a promising approach for optimizing water splitting performance in Ti‐based perovskite oxides doped with exsolvable metals. The broader implication is that this strategy can be extended to a wider range of material systems and electrocatalytic applications.

## 2 Results and Discussion

**Figure 1:**
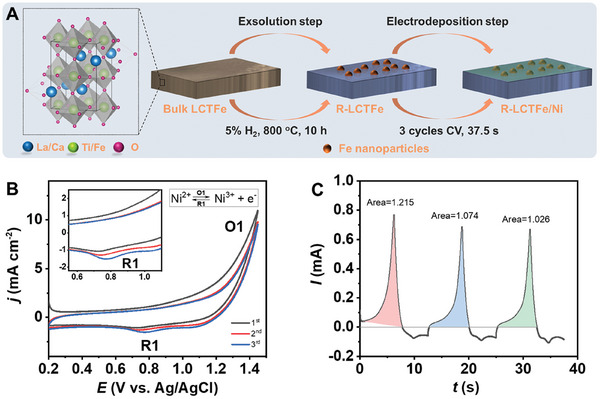
A) Schematic diagram of catalyst preparation using double E strategy. B) CVs for the electrodeposition of Ni^2+^. C) Integrogram for calculating the amount of transferred electrons during electrodeposition process.

The LCTFe perovskite oxides are synthesized through a convenient sol–gel approach, followed by a double E strategy processing to obtain R‐LCTFe/Ni. In **Figure** **1**A, the schematic diagram of the double E strategy is presented. Initially, iron nanoparticles are exsolved from the A‐site deficient LCTFe perovskites. The presence of A‐site deficiency serves as a driving force during the exsolution step.^[^ ^24^ ^]^ Following this, the electrodeposition step can be effortlessly achieved through the utilization of anodic electrochemical deposition techniques. Notably, a deposition time of only 37.5 s is achieved by employing just three cycles of cyclic voltammetry (CV).

The mechanism of the CV electrodeposition procedure is illustrated in Figure 1B. The current of oxidation peak O1 initiates at a potential of ≈0.8 V versus Ag/AgCl, which is attributed to the oxidation of Ni^2+^ to Ni^3+^. The corresponding reduction peak R1 is assigned to the reduction of Ni^3+^ to Ni^2+^.^[^ ^25^ ^]^ This process is followed by the reaction of Ni^3+^ ions with OH^−^ ions, which results in the formation of a thin and insoluble film of nickel oxy‐hydroxide, possibly identified as NiOOH, on the surface of R‐LCTFe.^[^ ^25^ ^]^ The entire process can be expressed through the following equations:^[^ ^25^ ^]^

(1) $$Ni^{2+}⇌Ni^{3+}+e^{-}$$

(2) $$Ni^{3+}+3OH^{-}\to \mathrm{NiOOH}↓+H_{2}O$$

The transferred electric charge (*Q)* during the procedure can be determined by summing the shaded areas in Figure 1C, and subsequently used to estimate the amount of substance (*n*) of electrodeposit nickel via Equation 3.

(3) $$n=\frac{Q}{e\cdot NA}$$

The calculated mole amount of nickel is 3.44 × 10^−8^ mol, corresponding to a mass of 2.02 × 10^−3^ mg, with a surface loading of 0.029 mg cm^−2^.

**Figure 2:**
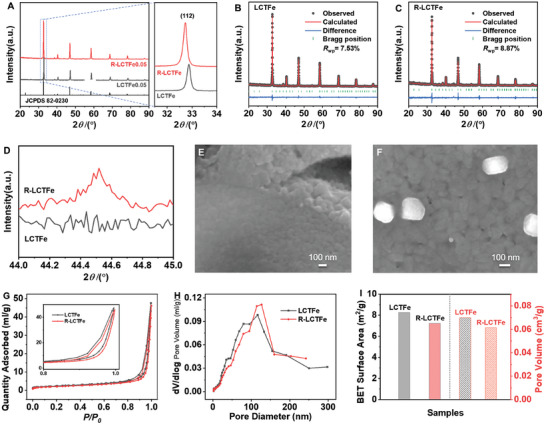
A) XRD patterns of LCTFe and R‐LCTFe with enlarged patterns in the 2*θ* range of 31° – 34°. Rietveld refinement of XRD data of B) LCTFe (*R*_wp_ = 7.53%) and C) R‐LCTFe (*R*_wp_ = 8.87%). D) Magnified XRD patterns of LCTFe and R‐LCTFe between 44° and 45° 2*θ*. SEM images of E) LCTFe and F) R‐LCTFe, showing their surface morphology. G) Nitrogen adsorption‐desorption isotherms, H) pore size distribution plots, and I) corresponding specific surface area and pore volume measurements for both LCTFe and R‐LCTFe, indicating their respective porosity characteristics.

To investigate the crystal structure of the as‐prepared LCTFe and R‐LCTFe samples, X‐ray diffraction (XRD) patterns are acquired and presented in **Figure** **2**A–C. The diffraction peaks observed in the LCTFe sample are assigned to the diffraction patterns of CaTiO_3_ (JCPDS No. 82–0230). Specifically, the diffraction peaks located at 32.7, 38.6, 40.4, 47.1, 54.2, 58.5, 68.7, 78.3, and 87.5° can be attributed to the (112), (211), (202), (004), (222), (312), (400), (116), and (044) lattice planes of the CaTiO_3_ crystal structure. Moreover, the substitution of La and Fe in the lattice of CaTiO_3_ occurs without the introduction of impurities, as evidenced by the Rietveld refinement of the XRD data in Figure 2B. The crystallographic parameters for both LCTFe and R‐LCTFe, as determined through refinement, are summarized in **Table** **1**.

**Table 1:** Structural parameters of LCTFe and R‐LCTFe obtained by crystallographic refinement.

Sample	Symmetry	Space group	Refined Cell parameters [Å]			Cell volume (Å^3^)
			*a*	*b*	*c*	
LCTFe	Orthorhombic P	Pbnm (62)	5.435(3)	5.4413(10)	7.694(3)	227.54(23)
R‐LCTFe	Orthorhombic P	Pbnm (62)	5.447(6)	5.4503(15)	7.707(6)	228.8(5)

It is noteworthy that the unit cell of LCTFe (5.435 × 5.4413 × 7.694, Table 1) is smaller than that for CaTiO_3_ (5.445 × 5.4708 × 7.7247, JCPDS No. 82–0230). This phenomenon suggests that lattice shrinkage may have resulted from the A‐site vacancy and the substitution of La and Fe in the CaTiO_3_ lattice. Furthermore, the XRD patterns of R‐LCTFe are almost identical to those of LCTFe, indicating that the main CaTiO_3_ structure of R‐LCTFe remains unchanged after the exsolution process, with a small expansion. The unit cell primarily compensates for B‐site loss by reducing the A‐site deficiency. The refined cell parameters presented in Table 1 confirm that both LCTFe and R‐LCTFe samples possess an orthorhombic structure with a space group of Pbnm. Particularly, the cell parameters of R‐LCTFe are slightly expanded in all three dimensions compared to those of LCTFe. These finding are in line with previous studies, which have reported that the reduction of smaller Ti^4+^ ions (0.605 Å) to larger Ti^3+^ ions (0.67 Å) on the B‐site can result in lattice expansion.^[^ ^16^ ^]^ Within the 2*θ* range of 44.4–44.6°, the observed peak in Figure 2D corresponds to the (110) reflections of zero‐valent Fe (JCPDS No. 87–0721), confirming that the exsolved Fe nanoparticles are in the metallic form.^[^ ^26^ ^]^

Scanning electron microscopy (SEM) images presented in Figure 2E,F illustrate the surface morphologies of LCTFe and R‐LCTFe. As depicted in Figure 2E, the surface of LCTFe consists of numerous integrated grains, with the shape of the grains barely distinguishable. In Figure 2F, the surface grains on R‐LCTFe closely resemble those on LCTFe. Exsolved nanoparticles are clearly anchored on the surface, ≈100–150 nm in size. The energy dispersive spectrometer (EDS) analysis presented in Figure S2, Tables S2 and S3 (Supporting Information) demonstrates the presence of the constituent elements La, Ca, Ti, Fe, and O in both LCTFe and R‐LCTFe samples, which is consistent with their expected chemical composition. Figure 2G–I displays the nitrogen adsorption and desorption isotherms and the associated physical properties, including pore size distribution, BET specific surface area, and pore volume for the samples under investigation. Based on the IUPAC classification, both samples exhibit Type IV adsorption isotherms, indicating the presence of mesoporous structures.^[^ ^27^ ^]^ The steep rise in the isotherms near P/P_0_ = 1 suggests the existence of macropores (pore size >50 nm), which is consistent with the pore size distribution plot in Figure 2H.^[^ ^27^ ^]^ It can be observed that the pore size distribution of the samples is centered ≈110 nm. Figure 2I reveals that LCTFe exhibits a higher BET surface area (8.2 m^2^ g^−1^) and pore volume (0.070 cm^3^ g^−1^) compared to R‐LCTFe (7.2 m^2^ g^−1^, 0.061 cm^3^ g^−1^).

The amount of exsolved Fe nanoparticles can be estimated using thermogravimetric analysis (TGA). TGA of R‐LCTFe (Figure S3, Supporting Information) revealed an overall weight gain of ≈0.68%. When the temperature reached ≈460 °C, the weight stabilized. This weight increase is primarily attributed to the re‐oxidation of Fe nanoparticles, leading to the formation of Fe_2_O_3_ nanoparticles.^[^ ^28^ ^]^ Therefore, the mass ratio of the exsolved Fe nanoparticles is estimated to be below 1.58%.

**Figure 3:**
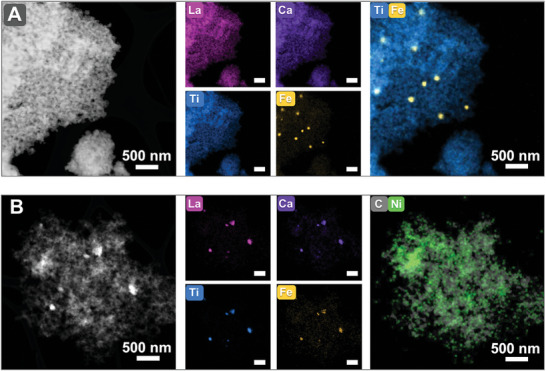
TEM and its corresponding elemental mapping images of A) R‐LCTFe powder sample and B) R‐LCTFe/Ni catalyst scraped from GCE.

To investigate the structure of the R‐LCTFe and R‐LCTFe/Ni catalysts on the glassy carbon electrode (GCE), we performed ex situ scanning electron microscopy (SEM) imaging, as shown in Figure S4 (Supporting Information). The images suggest that both catalysts are dispersed within a multihole conductive carbon matrix, with the only discernible distinction being the size of the dispersed R‐LCTFe particles. Additionally, we are able to detect the presence of electrodeposited Ni in the R‐LCTFe/Ni catalyst using EDS mapping, as shown in Figures S5 and S6 (Supporting Information). However, due to the thickness of the sample layer on the GCE, accurately determining the actual distribution of these elemental components is challenging. To overcome this limitation, TEM is utilized to obtain more detailed structural information about these samples. The uniform distribution of La, Ca, Ti, Fe, and O elements in the LCTFe catalyst is confirmed by the TEM EDS‐mapping results in Figure S7 (Supporting Information). These results further support the even doping of La and Fe in the CaTiO_3_ lattice structure. Following the exsolution step, TEM mapping images of R‐LCTFe in **Figures** **3**A and S8 (Supporting Information) reveal exsolved iron nanoparticles, providing direct evidence of successful exsolution from the parent perovskite. The size of these nanoparticles is detected to be ≈100 nm, which is consistent with SEM analysis.

To prepare the R‐LCTFe/Ni catalyst, we performed electrodeposition on a GCE modified with the R‐LCTFe catalyst. For microstructural analysis, the R‐LCTFe/Ni catalyst was removed from the electrode surface by scraping for TEM characterization. The TEM image and corresponding elemental maps of the as‐prepared R‐LCTFe/Ni catalyst are shown in Figure 3B, while the separate mapping images for C and Ni are provided in Figure S9 (Supporting Information).

The simultaneous detection of La, Ca, Ti, and Fe in the same region, as revealed by TEM mapping, strongly indicates that R‐LCTFe is well‐dispersed within the conductive carbon matrix. Additionally, the electrodeposited Ni predominantly adheres to the carbon support, suggesting that Ni was evenly distributed across the GCE surface before removal, aligning with our expectations. The uniform elemental distribution observed in TEM mapping further verifies that the electrodeposition process does not interfere with the exsolved Fe nanoparticles. These Fe nanoparticles remain stable before and after Ni deposition, demonstrating that the electrodeposited Ni interacts with both the conductive carbon and pre‐existing Fe sites without altering their positions or structures. Thus, the integrity of the exsolved Fe nanoparticles is maintained throughout the electrodeposition process.

Furthermore, the Raman spectra of both R‐LCTFe and R‐LCTFe/Ni catalysts on the GCE (Figure S10, Supporting Information) show nearly identical features. Peaks observed at 199, 236, 285, and 350 cm^−1^ are linked to the bending modes of O─Ti─O bonds, while the bands at 531 and 801 cm^−1^ are attributed to the asymmetric and symmetric vibrations of the TiO_6_ octahedral units, often described as “breathing” modes.^[^ ^29^, ^30^, ^31^, ^32^ ^]^ The peak ≈730 cm^−1^, attributed to Fe─O vibrations, also remains unchanged between the two samples.^[^ ^33^, ^34^ ^]^ The similarity in the Raman spectra suggests that the exsolved Fe nanoparticles are unaffected by the subsequent Ni electrodeposition, further confirming that the deposition process does not significantly impact the structural integrity of the exsolved particles.

**Figure 4:**
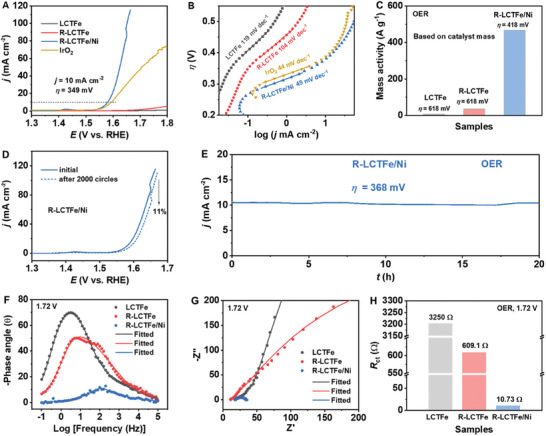
A) OER LSV curves of LCTFe, R‐LCTFe, R‐LCTFe/Ni and IrO_2_ in a 1.0 м KOH solution. B) Corresponding Tafel plots and C) OER mass activities based on catalyst mass at an overpotential of 418 mV for R‐LCTFe/Ni and 618 mV for LCTFe and R‐LCTFe, respectively. D) LSV curves of R‐LCTFe/Ni before and after 2000 cycles of CV. E) OER long‐term durability test of R‐LCTFe/Ni at *η* = 368 mV for 20 h in a 1.0 м KOH solution. F) EIS Bode plots, G) Nyquist plots and H) *R*_ct_ values of all three samples fitted from EIS data at 1.72 V versus RHE.

The use of the double E strategy in R‐LCTFe/Ni results in a significant enhancement in its performance for the OER in comparison to LCTFe. This is clearly demonstrated by the results in **Figure** **4**A, where the overpotential (*η*_10_) of R‐LCTFe/Ni at 10 mA cm^−2^ is only 349 mV in 1.0 м KOH, whereas LCTFe and R‐LCTFe cannot achieve 10 mA cm^−2^ even at *η* = 588 mV. In fact, the OER performance of R‐LCTFe/Ni exceeds that of the perovskite benchmark catalyst BSCF (*η*_10_ = 500 mV),^[^ ^35^, ^36^ ^]^ as well as the commercial precious metal catalysts RuO_2_ (*η*_10_ from 370 to 420 mV)^[^ ^35^, ^37^, ^38^, ^39^ ^]^ and IrO_2_ (*η*_10_ = 363 mV, which is consistent with reported literature).^[^ ^40^, ^41^, ^42^ ^]^ The superior performance of R‐LCTFe/Ni in OER can be attributed to its faster kinetics, as evidenced by the Tafel plots in Figure 4B. R‐LCTFe/Ni exhibits a significantly lower Tafel slope of 45 mV dec^−1^, which is much lower than the values obtained for LCTFe (119 mV dec^−1^) and R‐LCTFe (104 mV dec^−1^).

Moreover, the Tafel slope of R‐LCTFe/Ni is also lower than those reported for the perovskite benchmark catalyst BSCF, and compares favorably with commercial precious metal catalysts RuO_2_ and IrO_2_, as evidenced by our measurements (Tafel slope of IrO_2_: 44 mV dec^−1^) and previous literature.^[^ ^35^, ^36^, ^37^, ^38^, ^39^, ^40^, ^41^, ^42^ ^]^ The lower Tafel slope value for R‐LCTFe/Ni indicates a faster reaction rate, which is consistent with its superior OER activity. Figure S11 (Supporting Information) shows that the onset potentials of GCE/Ni and LCTFe/Ni are significantly shifted to more positive potential regions, and the current densities at the same overpotentials are decreased, when compared to R‐LCTFe/Ni. This indicates a degradation in the OER activity of GCE/Ni and LCTFe/Ni. Additionally, the higher Tafel slopes of GCE/Ni and LCTFe/Ni compared to R‐LCTFe/Ni suggest a slower reaction rate. These findings highlight the significance of the double E strategy and the synergistic cooperation between exsolved Fe nanoparticles and electrodeposited Ni compounds in enhancing the OER activity of R‐LCTFe/Ni.

As depicted in Figure 4C and Figure S12 (Supporting Information), R‐LCTFe/Ni exhibits the highest mass activity. Specifically, the mass activity of R‐LCTFe/Ni (466 A g^−1^) at *η* = 418 mV is ≈66 and 13 times higher than that of LCTFe (7 A g^−1^) and R‐LCTFe (36 A g^−1^) at *η* = 618 mV, respectively. This observation suggests a significant enhancement in the OER activity of R‐LCTFe/Ni after implementing the double E strategy. Additionally, the mass activity of R‐LCTFe/Ni based on the total mass of Fe and Ni at *η* = 348 mV reaches up to 2500 A g^−1^, indicating an exceptional utilization rate of nickel‐iron atoms (Figure S13, Supporting Information). The electrochemically active surface area (ECSA) of LCTFe, R‐LCTFe, and R‐LCTFe/Ni, determined from the corresponding CV curves (Figure S15, Supporting Information), is presented in Figure S14 (Supporting Information). Interestingly, they exhibit similar ECSA values of ≈5 cm^2^, suggesting that their activity is not solely governed by ECSA. Furthermore, the specific activity (SA) normalized to ECSA is calculated to evaluate the intrinsic activity of the catalysts. As shown in Figure S16 (Supporting Information), the SA of R‐LCTFe/Ni significantly surpasses that of LCTFe and R‐LCTFe, indicating a distinct enhancement in intrinsic activity achieved through the double E strategy. The remarkable OER activity of R‐LCTFe/Ni is comparable to various well‐known and highly active perovskite‐based OER catalysts reported in literatures (Table S1, Supporting Information). In addition to activity, stability is also a crucial factor for practical applications. To evaluate the stability, continuous cycling is performed for 2000 cycles at a scan rate of 100 mV s^−1^ within the OER potential window of 1.32 to 1.82 V versus RHE. The obtained LSV curve at the end of cycling demonstrates only a minor decrease in current density (Figure 4D), indicating the excellent stability of R‐LCTFe/Ni. Additionally, R‐LCTFe/Ni demonstrates superior stability by maintaining a current density above 10 mA cm^−2^ throughout a 20 h constant potential electrolysis test (*η* = 368 mV) as shown in Figure 4E, reaffirming its robustness once again.

The OER kinetics of LCTFe, R‐LCTFe, and R‐LCTFe/Ni were further evaluated using electrochemical impedance spectroscopy (EIS). Figure 4F displays the EIS Bode plots, revealing that the characteristic frequency associated with OER of R‐LCTFe/Ni is observed at ≈200 Hz at 1.72 V versus RHE. However, the small peak in the frequency range of 100–200 Hz for R‐LCTFe is attributed to the surface double‐layer capacitance (DLC).^[^ ^43^ ^]^ Importantly, the characteristic frequency for OER of LCTFe and R‐LCTFe is significantly lower (≈5 Hz) than that of R‐LCTFe/Ni, indicating a much slower reaction rate. The corresponding Nyquist plots in Figure 4G demonstrate that R‐LCTFe/Ni exhibits a significantly lower charge transfer resistance (*R*_ct_), suggesting a faster electron transfer rate during the OER process. Figure 4H and Table S4 (Supporting Information) display the *R*_ct_ values for LCTFe, R‐LCTFe, and R‐LCTFe/Ni, which are 3250, 609.1, and 10.73 Ω, respectively. These *R*_ct_ values are derived from fitting the data to the Equivalent Circuit (EC) model detailed in Figure S17 (Supporting Information). Within the model, *R*_s_ indicates the solution resistance. *R*_a_, appearing as a semicircle in the high‐frequency region of the Nyquist plot, represents the internal charge transfer resistance.^[^ ^44^ ^]^ *R*_ct_ reflects the reaction kinetics at the electrode surface as the charge transfer resistance. The constant phase element (CPE) accounts for non‐ideal capacitive behavior, describing complex impedance in electrochemical systems. These results elucidate the underlying reason for the superior performance of R‐LCTFe/Ni in OER.

Oxygen vacancies are generated during the synthesis of R‐LCTFe. However, a comparative analysis of the catalytic activities between R‐LCTFe and LCTFe reveals that the increase in activity after reduction treatment is quite limited and is predominantly attributed to the Fe nanoparticles that are exsolved and anchored onto the perovskite surface. Furthermore, the integration of these Fe nanoparticles with Ni leads to the formation of new active sites, significantly enhancing the activity of R‐LCTFe/Ni. This suggests that the role of oxygen vacancies in enhancing the performance of R‐LCTFe/Ni may be even more limited.

**Figure 5:**
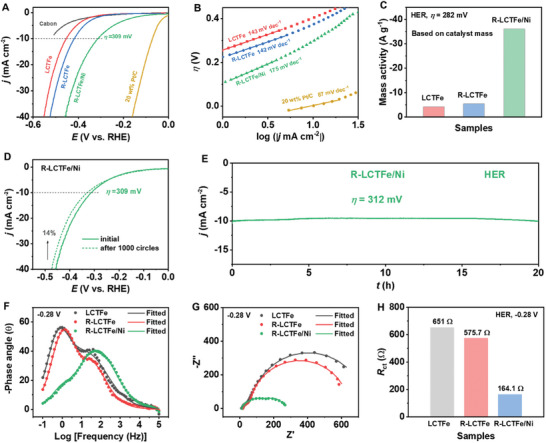
A) HER LSV curves of carbon, LCTFe, R‐LCTFe, R‐LCTFe/Ni and 20 wt.% Pt/C in a 1.0 м KOH solution. B) Corresponding Tafel plots and C) HER mass activities based on catalyst mass at an overpotential of 282 mV. D) LSV curves of R‐LCTFe/Ni before and after 1000 cycles of CV. E) HER long‐term durability test of R‐LCTFe/Ni at *η* = 312 mV for 20 h in a 1.0 м KOH solution. F) EIS Bode plots, G) Nyquist plots and H) *R*_ct_ values of all three samples fitted from EIS data at −0.28 V versus RHE.

The HER catalytic performance of carbon, LCTFe, R‐LCTFe, R‐LCTFe/Ni, and 20 wt.% Pt/C is evaluated under alkaline conditions in a 1.0 м KOH electrolyte. **Figure** **5**A demonstrates that the reference sample carbon exhibits negligible activity for HER. However, the implementation of the double E strategy results in enhanced HER activity for R‐LCTFe/Ni, as indicated by lower overpotentials required to achieve a current density of 10 mA cm^−2^. The overpotentials follow the sequence: 20 wt.% Pt/C (71 mV) < R‐LCTFe/Ni (309 mV) < R‐LCTFe (416 mV) < LCTFe (452 mV). Figure 5B reveals that both LCTFe and R‐LCTFe display lower Tafel slopes (143 and 142 mV dec^−1^, respectively) compared to R‐LCTFe/Ni (175 mV dec^−1^). It is worth noting that LCTFe and R‐LCTFe necessitated higher overpotentials to facilitate the release of hydrogen. In the field of HER, it is widely acknowledged that the Tafel slopes of 120, 40, and 30 mV dec^−1^ correspond to the Volmer, Heyrovsky, and Tafel determining rate steps, respectively.^[^ ^45^ ^]^ In this study, the observed Tafel slopes for all our synthesized samples exceed 120 mV dec^−1^, including the 20 wt.% Pt/C catalyst, which achieves 87 mV dec^−1^. This phenomenon is likely due to the inhibition and deactivation of active sites by strongly adsorbed hydrogen (H_up_) under alkaline conditions, indicating higher resistance for HER in alkaline environments compared to acidic ones.^[^ ^46^ ^]^ This adsorption impedes the formation of weakly adsorbed hydrogen (H_op_), which is an intermediate in the HER process.^[^ ^47^ ^]^ Tafel slopes exceeding 120 mV dec^−1^ are frequently associated with the Volmer rate‐determining step; however, it should be noted that such slopes can arise from either the Volmer or the Heyrovsky rate‐determining steps, indicating potential variability in the underlying processes.^[^ ^45^, ^48^, ^49^ ^]^ The higher Tafel slopes observed for R‐LCTFe/Ni, in comparison to R‐LCTFe and LCTFe in the HER, can be attributed to changes in mass transport properties and hydrogen adsorption kinetics resulting from the incorporation of Ni into the catalyst structure. These modifications may lead to increased Tafel slopes, even if the intrinsic catalytic activity is enhanced.

To further validate the effectiveness of the double E strategy and the synergistic cooperation between exsolved Fe nanoparticles and electrodeposited Ni compounds in enhancing the performance of the HER, a comparison is made among GCE/Ni, LCTFe/Ni, and R‐LCTFe/Ni, as shown in Figure S18 (Supporting Information). The results are consistent with the findings from the OER analysis, characterized by a lower onset potential, reduced overpotential at −10 mA cm^−2^, and a lower Tafel slope observed for R‐LCTFe/Ni in comparison to GCE/Ni and LCTFe/Ni. Furthermore, as depicted in Figure 5C and Figure S19 (Supporting Information), R‐LCTFe/Ni exhibits a higher mass activity (36 A g^−1^) at *η* = 282 mV, which is 8.8‐fold higher than LCTFe (4.1 A g^−1^) and 6.7‐fold higher than R‐LCTFe (5.4 A g^−1^), respectively. Additionally, the improved specific activity of R‐LCTFe/Ni, as indicated in Figure S20 (Supporting Information), suggests an enhanced intrinsic activity. The LSV curve obtained after subjecting R‐LCTFe/Ni to 1000 CV cycles (Figure 5D) demonstrates minimal loss in electrochemical performance compared to the initial curve, thus indicating the stable electrocatalytic performance of R‐LCTFe/Ni for the HER. The 20 h chronoamperometric curve shown in Figure 5E further illustrates the excellent stability of R‐LCTFe/Ni for the HER under alkaline conditions.

**Figure 6:**
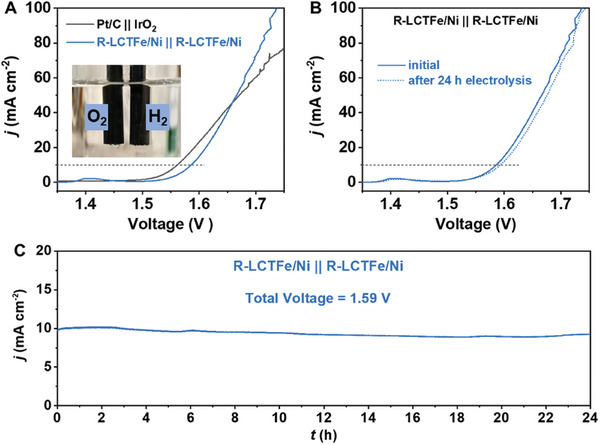
A) Overall water splitting LSV curves of R‐LCTFe/Ni || R‐LCTFe/Ni and Pt/C || IrO_2_ in 1.0 м KOH solution. B) Overall water splitting LSV curves of R‐LCTFe/Ni || R‐LCTFe/Ni before and after a 24‐h chronoamperometric test. C) Long‐term durability test of R‐LCTFe/Ni for overall water splitting at 1.59 V for 24 h in 1.0 м KOH solution.

The findings demonstrate that R‐LCTFe/Ni functions as an effective and stable bifunctional catalyst for both OER and HER in alkaline environments. To explore its overall water‐splitting capability, a two‐electrode configuration was constructed with R‐LCTFe/Ni used as both the cathode and anode (inset in **Figure** **6**A). This setup was tested in an alkaline solution, where the catalyst achieved a water‐splitting current density of 10 mA cm^−2^ at 1.58 V, with performance comparable to that of previously reported nonprecious metal catalysts.^[^ ^50^, ^51^, ^52^, ^53^ ^]^ Figure 6B displays LSV data recorded before and after a 24‐h durability test, with minimal performance degradation. Moreover, as illustrated in Figure 6C, the chronoamperometric measurement confirmed that R‐LCTFe/Ni maintained a stable current density of ≈10 mA cm^−2^ at 1.59 V over the 24‐h period. These results highlight R‐LCTFe/Ni's promising application for sustained water‐splitting in alkaline solutions.

The Bode plots depicted in Figure 5F exhibit discernible peaks in the high‐frequency range (30–50 Hz) for both LCTFe and R‐LCTFe, aligning with the first small semicircle observed in the Nyquist plots of Figure 5G. This behavior can be attributed to the inherent characteristics of the electrodes,^[^ ^54^, ^55^, ^56^ ^]^ which are influenced by the diverse catalyst materials employed. In contrast, the characteristic frequency peak associated with the HER activity of R‐LCTFe/Ni is situated at ≈200 Hz, significantly higher than the values observed for LCTFe and R‐LCTFe (≈1 Hz). This disparity suggests a notably accelerated reaction rate for R‐LCTFe/Ni, highlighting its enhanced performance in the HER process. The second semicircle observed in the Nyquist plots of Figure 5G corresponds to the lower frequency peak for the HER of LCTFe and R‐LCTFe in Figure 5F, representing the charge transfer resistance (*R*_ct_).^[^ ^57^, ^58^ ^]^ The *R*_ct_ values for LCTFe, R‐LCTFe, and R‐LCTFe/Ni are depicted in Figure 5H and detailed in Table S5 (Supporting Information). These values are derived from fitting the corresponding EIS data for the HER, utilizing the EC shown in Figure S17 (Supporting Information). It is apparent that R‐LCTFe/Ni demonstrates a lower *R*_ct_ compared to LCTFe and R‐LCTFe, indicating a more efficient charge transport mechanism during the electrochemical HER process.

In R‐LCTFe/Ni, pinpointing the precise active sites within this complex system, which comprises exsolved Fe nanoparticles, deposited Ni, and the parent perovskite, proves challenging. Unlike single‐crystal systems where facets with superior catalytic activity can be easily identified, the disorderly mixture of elements and facets here complicates this task. However, comparative experimental data indicate that the enhanced catalytic performance of R‐LCTFe/Ni arises from a synergistic interaction between exsolved Fe nanoparticles and electrodeposited Ni. Specifically, the comparison between LCTFe and R‐LCTFe highlights the catalytic enhancement due to exsolved Fe nanoparticles. Moreover, based on our previous research, the contribution of LCT to OER catalytic activity can be effectively ruled out.^[^ ^23^ ^]^ Additionally, the comparison between R‐LCTFe and R‐LCTFe/Ni demonstrates a substantial increase in activity with the latter, emphasizing the role of electrodeposited Ni. Finally, comparisons among LCTFe/Ni, R‐LCTFe, and R‐LCTFe/Ni show that while Ni deposition alone improves LCTFe's activity, it is considerably less effective than the combination found in R‐LCTFe/Ni. Thus, the catalytic active sites are inferred to consist of both exsolved Fe nanoparticles and electrodeposited Ni.

**Figure 7:**
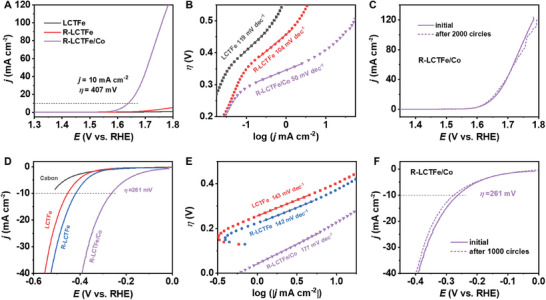
A) OER LSV curves of LCTFe, R‐LCTFe, and R‐LCTFe/Co in a 1.0 м KOH solution. B) Corresponding Tafel plots and C) LSV curves of R‐LCTFe/Co before and after 2000 cycles of CV. D) HER LSV curves of carbon, LCTFe, R‐LCTFe, and R‐LCTFe/Co in a 1.0 м KOH solution. E) Corresponding Tafel plots and F) LSV curves of R‐LCTFe/Co before and after 1000 cycles of CV.

To assess the transferability of the double E strategy, we substituted Co for Ni in the electrodeposition step. The activity results are presented in **Figure** **7** and Figures S20 and S21 (Supporting Information). As illustrated in Figure 7A and R‐LCTFe/Co demonstrates a significantly reduced overpotential (407 mV) at 10 mA cm^−2^ and a higher catalytic current density compared to LCTFe and R‐LCTFe, indicating superior OER activity. Figure 7B displays the Tafel plots that provide insights into the OER kinetics. Notably, R‐LCTFe/Co exhibits a smaller Tafel slope (50 mV dec^−1^) compared to LCTFe (119 mV dec^−1^) and R‐LCTFe (104 mV dec^−1^), suggesting enhanced effectiveness in catalyzing the OER. Figure S21 (Supporting Information) presents the OER LSV and Tafel comparisons among GCE/Co, LCTFe/Co, and R‐LCTFe/Co. These results further confirm the superior OER performance and faster OER rates of R‐LCTFe/Co, underscoring the significance of the double E strategy in enhancing the electrocatalytic activity for OER. Furthermore, the stability of R‐LCTFe/Co during the OER process is evaluated (Figure 7C). Notably, after undergoing 2000‐cycle continuous CV scans, the current of R‐LCTFe/Co exhibited negligible changes, indicating its high stability.

Moreover, the HER activity of LCTFe, R‐LCTFe, and R‐LCTFe/Co is assessed in a 1.0 м KOH solution at room temperature. Figure 7D displays their respective LSV curves, demonstrating that R‐LCTFe/Co exhibited a lower overpotential (261 mV) relative to LCTFe and R‐LCTFe at a current density of −10 mA cm^−2^, indicating improved HER activity resulting from the implementation of the double E strategy. Notably, similar to R‐LCTFe/Ni, R‐LCTFe/Co displayed a higher Tafel slope (177 mV dec^−1^) compared to LCTFe (143 mV dec^−1^) and R‐LCTFe (142 mV dec^−1^) as shown in Figure 7E. However, it is worth mentioning that LCTFe and R‐LCTFe required higher overpotentials to facilitate the release of hydrogen. Figure S22 (Supporting Information) further demonstrates that the overpotential for R‐LCTFe/Co at each current density is lower compared to GCE/Co and LCTFe/Co, and R‐LCTFe/Co displays a smaller Tafel slope than GCE/Co and LCTFe/Co, indicating superior HER catalytic activity and faster HER rate among the studied catalysts. The stability of R‐LCTFe/Co during HER is evaluated by conducting LSV measurements after 1000‐cycle continuous CV scans, which shows negligible changes in current density (Figure 7F), suggesting good stability. Cumulatively, these findings provide evidence for the effectiveness and transferability of the double E strategy in enhancing the catalytic performance of Ti‐based perovskite materials for both OER and HER.

## 3 Conclusion

In summary, we have successfully developed a double E strategy in Ti‐based perovskite materials to enhance the electrocatalytic performance for both OER and HER. R‐LCTFe/Ni exhibits high activity and long‐term stability in facilitating both processes. This exceptional catalytic performance can be attributed to the more efficient charge transport mechanism in R‐LCTFe/Ni and the synergistic cooperation between exsolved Fe nanoparticles and electrodeposited Ni. Furthermore, a 24‐h overall water‐splitting test of R‐LCTFe/Ni demonstrates its promising potential for sustained water‐splitting in alkaline solutions. Additionally, the transferability of the double E strategy has been validated through the development of R‐LCTFe/Co catalyst. These findings open up new possibilities for designing and developing low cost bifunctional electrocatalysts based on perovskite oxides for simultaneous OER and HER applications.

## Conflict of Interest

The authors declare no conflict of interest.

## Supporting information



Supporting Information

## Data Availability

The research data underpinning this publication can be accessed at https://doi.org/10.17630/dc31454b‐5bfc‐43af‐b1ea‐709a252ca070.
