# Supplementary material for: Combined Exsolution and Electrodeposition Strategy for Enhancing Electrocatalytic Activity of Ti‐Based Perovskite Oxides in Oxygen and Hydrogen Evolution Reactions
Source: Adv Sci (Weinh). 2024 Dec 20;12(6):2410535. doi: 10.1002/advs.202410535 (PMC11809394; doi:10.1002/advs.202410535)
Supplement: Supplementary file 1 — Supporting Information [file ADVS-12-2410535-s001.docx]

Supporting information

**Combined Exsolution and Electrodeposition Strategy for Enhancing Electrocatalytic Activity of Ti-Based Perovskite Oxides in Oxygen and Hydrogen Evolution Reactions**

Shangshang Zuo, Chenchen Wang, Zhi Xia, Jiaxin Ding, Aaron B. Naden, and John T. S. Irvine*

S. Zuo, C. Wang, Z. Xia, J. Ding, A. B. Naden, J. T. S. Irvine

School of Chemistry, University of St Andrews, St Andrews, Fife KY16 9ST, UK

E-mail: jtsi@st-andrews.ac.uk

**Experimental details**

1. **Synthesis of La_0.25_Ca_0.65_Ti_0.95_Fe_0.05_O_3_ (LCTFe)**

The perovskite LCTFe was synthesized using the sol-gel method. 0.01 mol of a 50 wt % solution of Titanium (IV) bis(ammonium lactato) dihydroxide in H_2_O and 15 mL ethylene glycol were mixed together under magnetic stirring. Next, 10 g citric acid was added to the solution along with 5 mL of deionized water to facilitate dissolution. Stoichiometric amounts of La(NO_3_)_3_·6H_2_O, Ca(NO_3_)_2_·4H_2_O and Fe(NO_3_)_3_·6H_2_O were dissolved in deionized water and slowly added to the mixture while heating and stirring continuously. The resulting solution was magnetically stirred and heated on a hot plate at 200°C until it became a viscous gel. The gel was carbonized at 300°C for initial treatment, and the residue obtained was calcined at 550°C for 2 hours to eliminate any remaining carbon. Finally, LCTFe samples were obtained by calcining the precursors in a muffle furnace at 1000°C for 8 hours.

1. **Double E strategy**

The Double E strategy involved two distinct steps, exsolution and electrodeposition. During the exsolution step, iron nanoparticles were exsolved from the bulk LCTFe substrate. The subsequent electrodeposition step was performed on the prepared working electrode. Further details are provided below. Exsolution step: LCTFe samples were subjected to a 10-hour sintering process in a tubular furnace, under a 5% H_2_/N_2_ atmosphere at 800°C, which resulted in the production of R-LCTFe samples. Preparation of the working electrode: The catalyst ink was created using 6 mg of R-LCTFe, 6 mg of conductive carbon, 0.1 mL of Nafion (5 wt%), and 1.4 mL of ethanol, which were subjected to ultrasonic processing for 2 hours. The ink was then dropped onto a glassy carbon electrode (GCE) with a diameter of 3 mm and air-dried, resulting in a catalyst mass loading (R-LCTFe) of approximately 0.0144 mg, with a surface loading of 0.21 mg cm^−2^. Electrodeposition step: Electrodeposition was carried out through cyclic voltammetry (CV) in a conventional three-electrode setup with a graphite rod as the counter electrode, Ag/AgCl as the reference electrode, and GCE loading with R-LCTFe as the working electrode as previously stated. The potential was scanned under the range of 0.2 to 1.45 V versus Ag/AgCl by applying a scan rate of 200 mV s^−1^ for a total of three cycles in an electrolyte of 0.1 м KCl containing 0.0015 mol Ni^2+^. This entire process was completed in only 37.5 seconds.

1. **Material Characterizations**

Powder X-ray diffraction (XRD) measurements were conducted on a PANalytical Empyrean diffractometer with Cu-Kα1 radiation and a zero-background Si substrate holder to minimize background noise. Scanning electron microscopy (SEM, JEOL JSM-IT800) and transmission electron microscopy (TEM, FEI Titan Themis) were utilized to examine the morphological properties. Additionally, energy-dispersive spectroscopy (EDS) was employed to analyze the elemental composition. The BET specific surface area was measured using a Micromeritics TriStar II 3020 instrument through nitrogen adsorption-desorption tests. The pore size distribution was analyzed with the Barrett-Joyner-Halenda (BJH) model.

1. **Electrochemical Measurements**

The electrochemical measurements were conducted on an AMETEK VersaSTAT 3 electrochemical workstation at room temperature in a standard three-electrode configuration with a 1.0 м KOH electrolyte. The three-electrode system consisted of a glassy carbon electrode (GCE) modified with catalyst serving as the working electrode, a graphite rod functioning as the counter electrode, and a double junction Ag/AgCl electrode acting as the reference electrode. The polarization curves for both OER and HER were measured using linear sweep voltammetry (LSV) and corrected for ohmic drop (iR). The electrochemically active surface area (ECSA) was estimated by performing CV with scanning rates ranging from 10 to 200 mV s^−1^ in a potential window from 0.1 to 0.2 V *vs.* Ag/AgCl, where no faradaic current was detected. The stability of OER and HER was evaluated by subjecting them to 2,000 (1,000) cycles of CV from 1.32 to 1.82 V (0.02 to −0.5 V) *vs.* RHE. Electrochemical impedance spectroscopy (EIS) measurements were collected at various voltages over a frequency range from 10,000 to 0.1 Hz.

The Ag/AgCl reference electrode was calibrated against a reversible hydrogen electrode (RHE) in a standard three-electrode configuration, using Pt foil as both the working and counter electrodes with an H_2_ gas inlet.^[1, 2]^ Cyclic voltammetry (CV) was conducted within the potential range for hydrogen electrocatalysis at a scan rate of 1 mV s^−1^ in 1 м KOH. As illustrated in Figure S1, the CV curve for HER showed two voltage intercepts at zero current. The average of these intercepts provided a calibrated potential of −1.018 V for the Ag/AgCl electrode.

Thus, in this study, all measured potentials were converted to the reversible hydrogen electrode (RHE) scale using the following equation: $E_{RHE}= E_{Ag/AgCl}+ 1.018 V.$ The overpotential (*η*) was calculated as $\eta= E_{RHE}-E_{equilibrium}$.


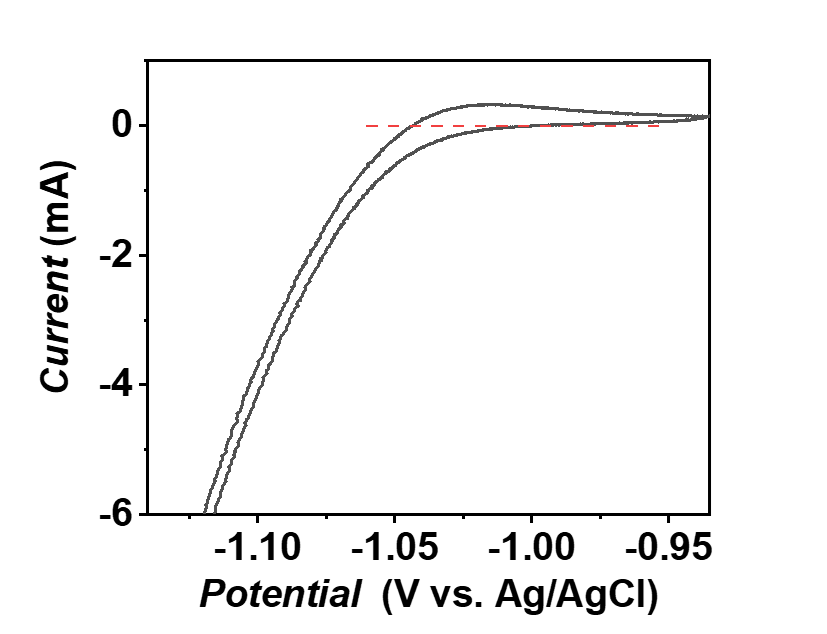


Figure S1. Calibration of Ag/AgCl electrode in H_2_-saturated 1 м KOH.


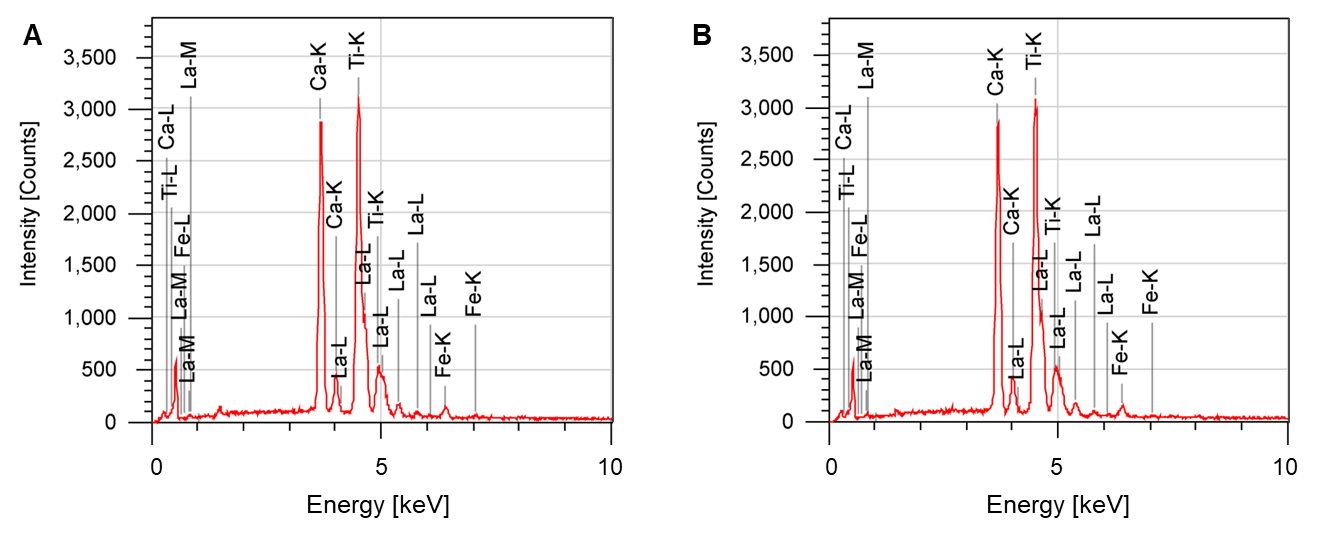


Figure S2. EDS patterns of (A) LCTFe and (B) R-LCTFe.


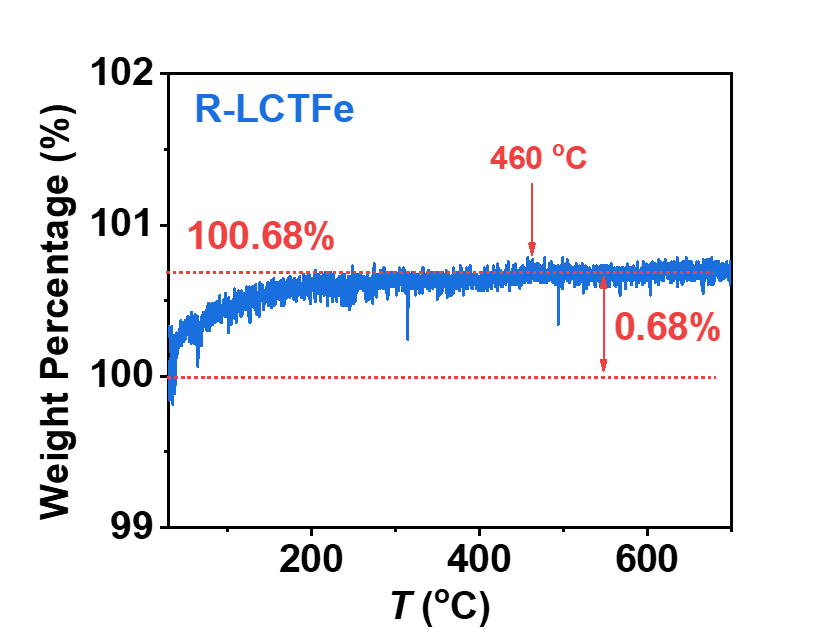


Figure S3. Thermogravimetric analysis (TGA) of R-LCTFe to 700 ^o^C in the air.


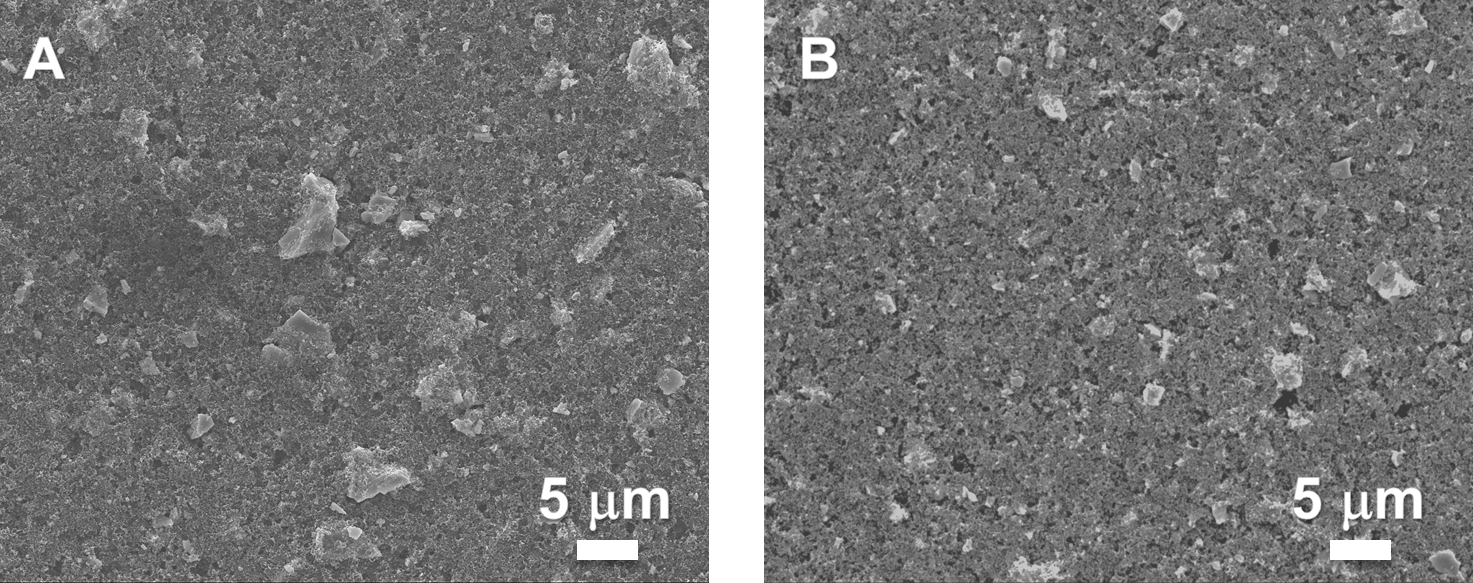


Figure S4. Ex-situ SEM images of (A) R-LCTFe and (B) R-LCTFe/Ni catalyst on a glassy carbon electrode.


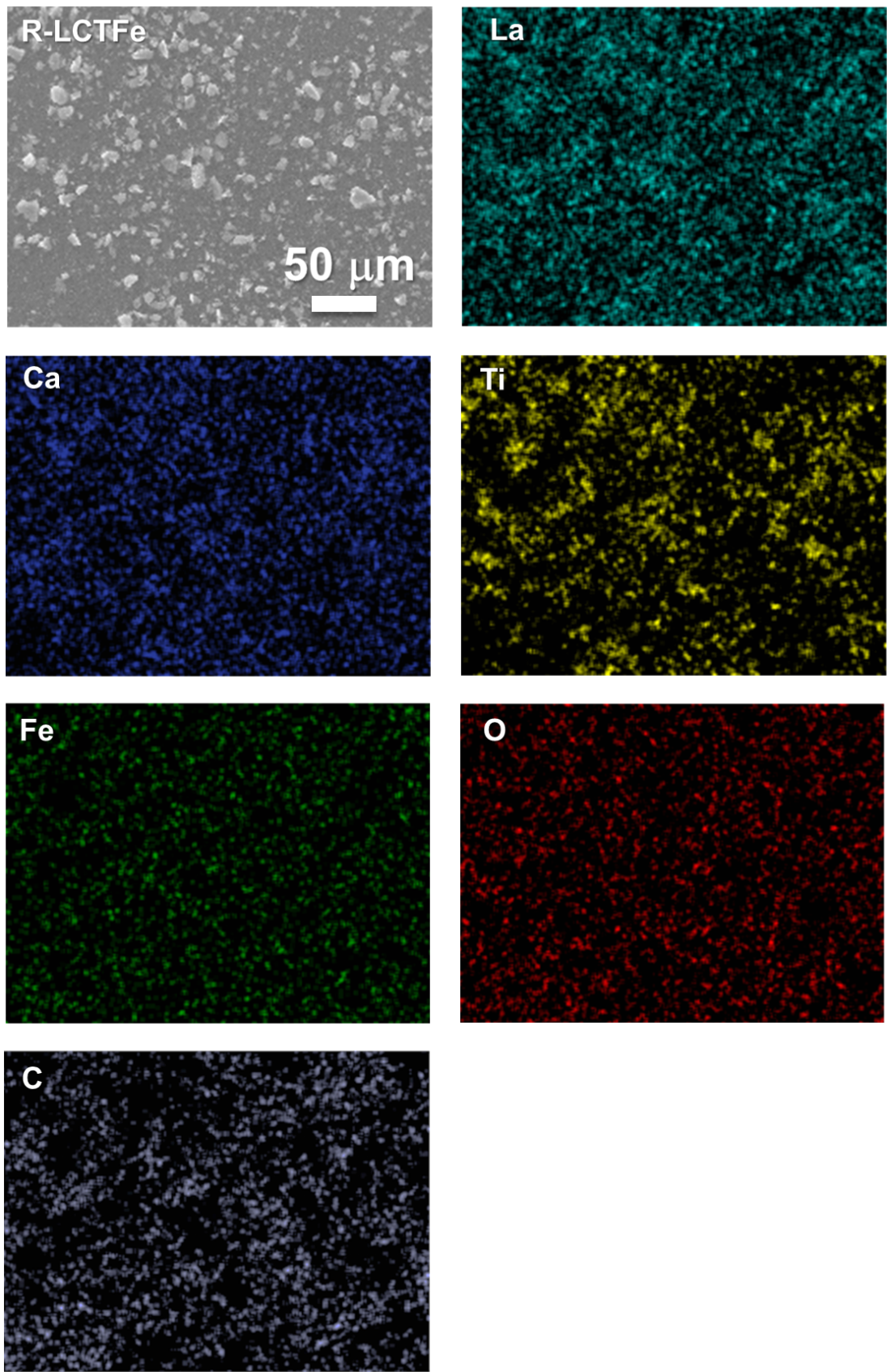


Figure S5. Ex-situ SEM image of R-LCTFe catalyst on a glassy carbon electrode and corresponding elemental mapping images (La, Ca, Ti, Fe, O, and C).


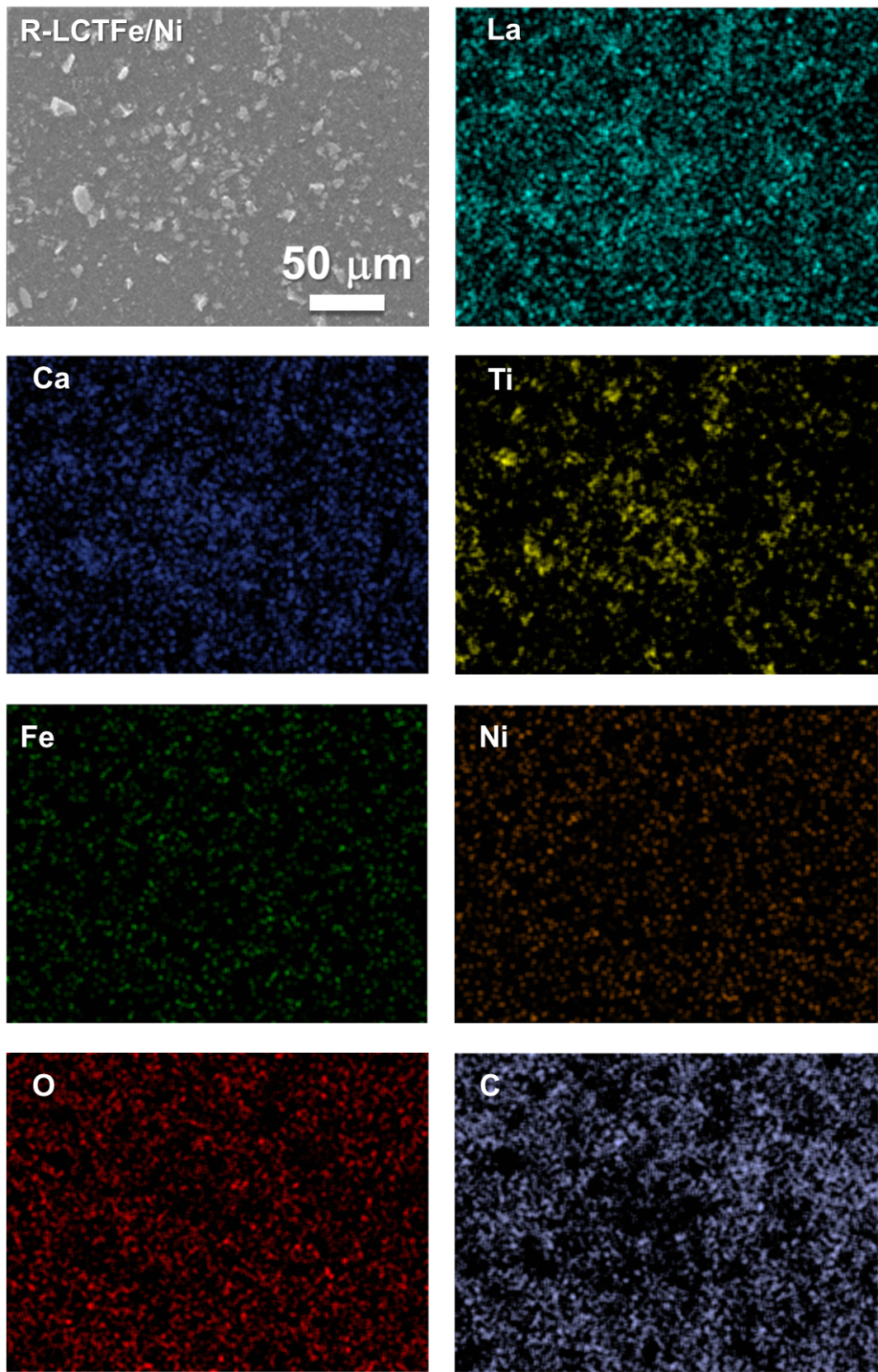


Figure S6. Ex-situ SEM image of R-LCTFe/Ni catalyst on a glassy carbon electrode and corresponding elemental mapping images (La, Ca, Ti, Fe, Ni, O, and C).


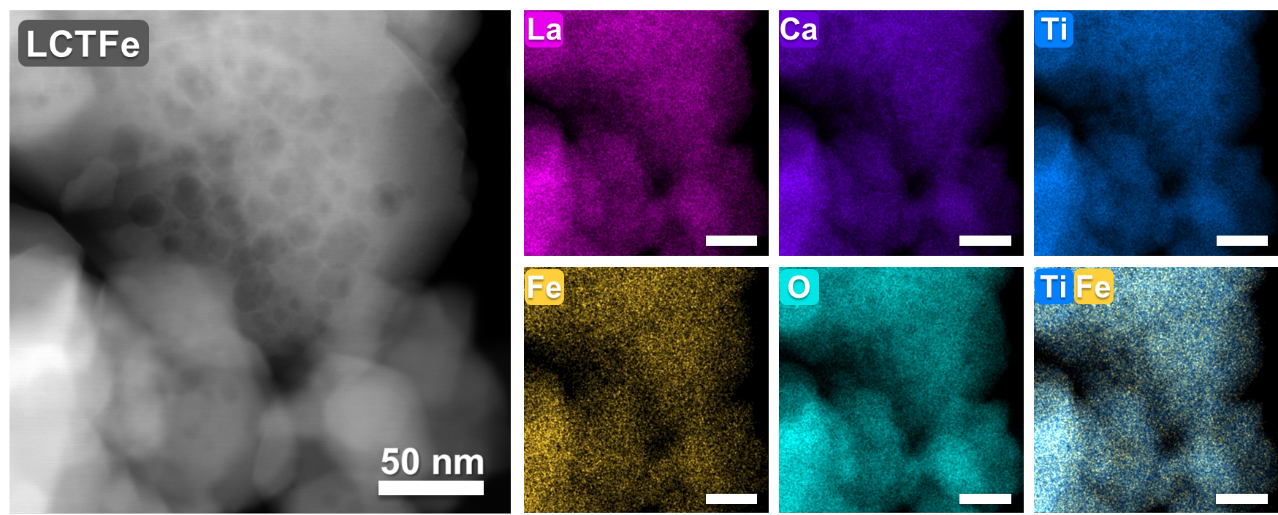


Figure S7. TEM image of LCTFe and corresponding elemental mapping images.


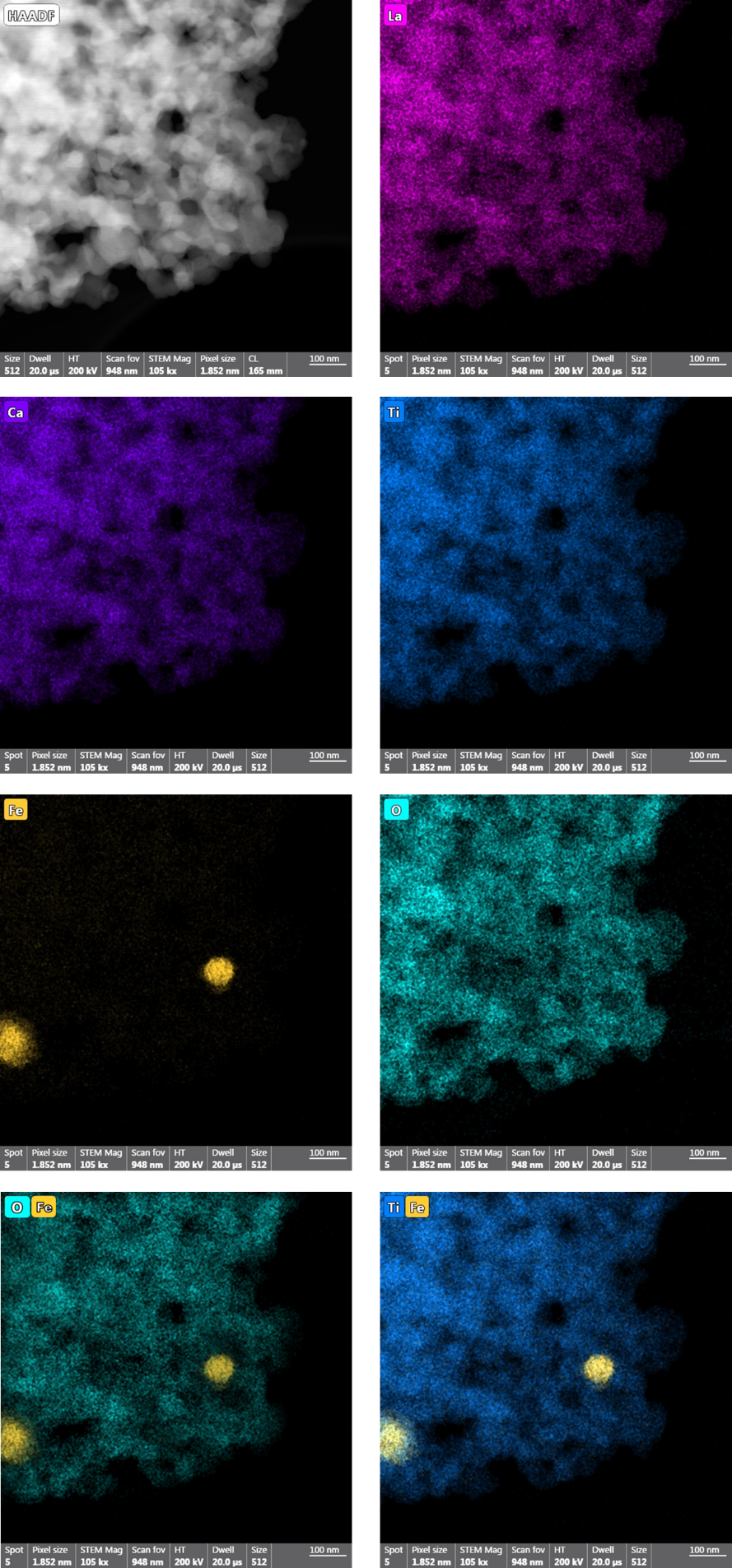


Figure S8. TEM image of R-LCTFe and corresponding elemental mapping images.


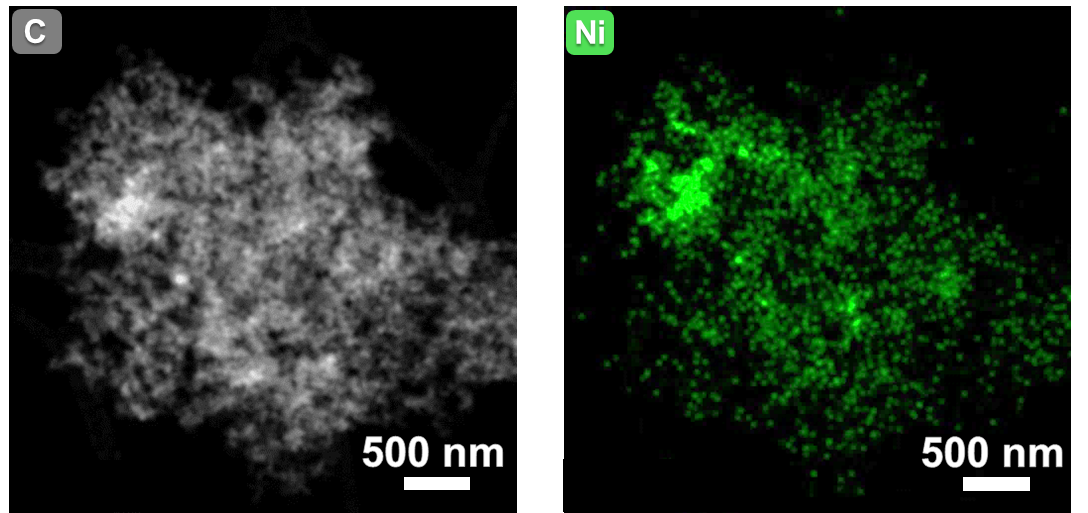


Figure S9. TEM elemental mapping images of carbon (grey) and nickel (green) for the R-LCTFe/Ni catalyst scraped from a glassy carbon electrode.


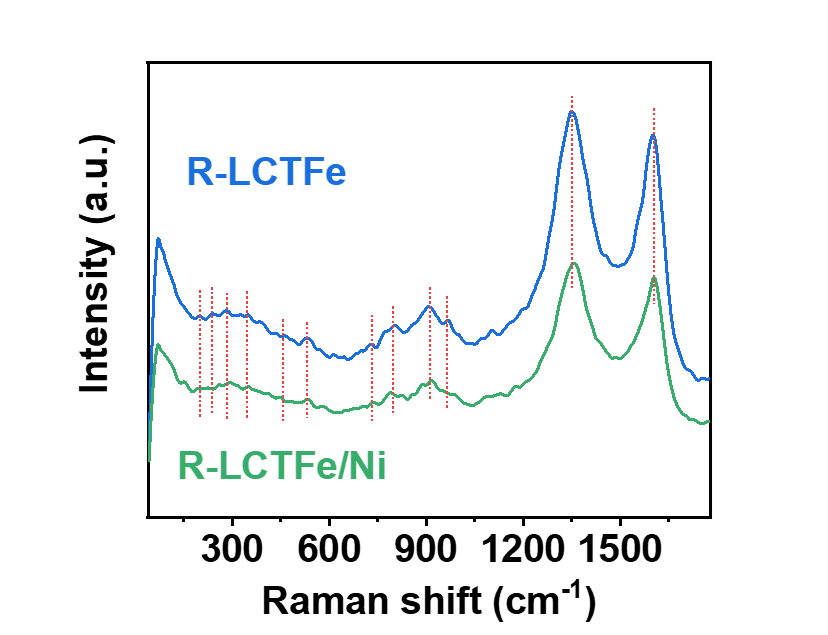


Figure S10. Raman spectra of R-LCTFe and R-LCTFe/Ni catalysts on a glassy carbon electrode.


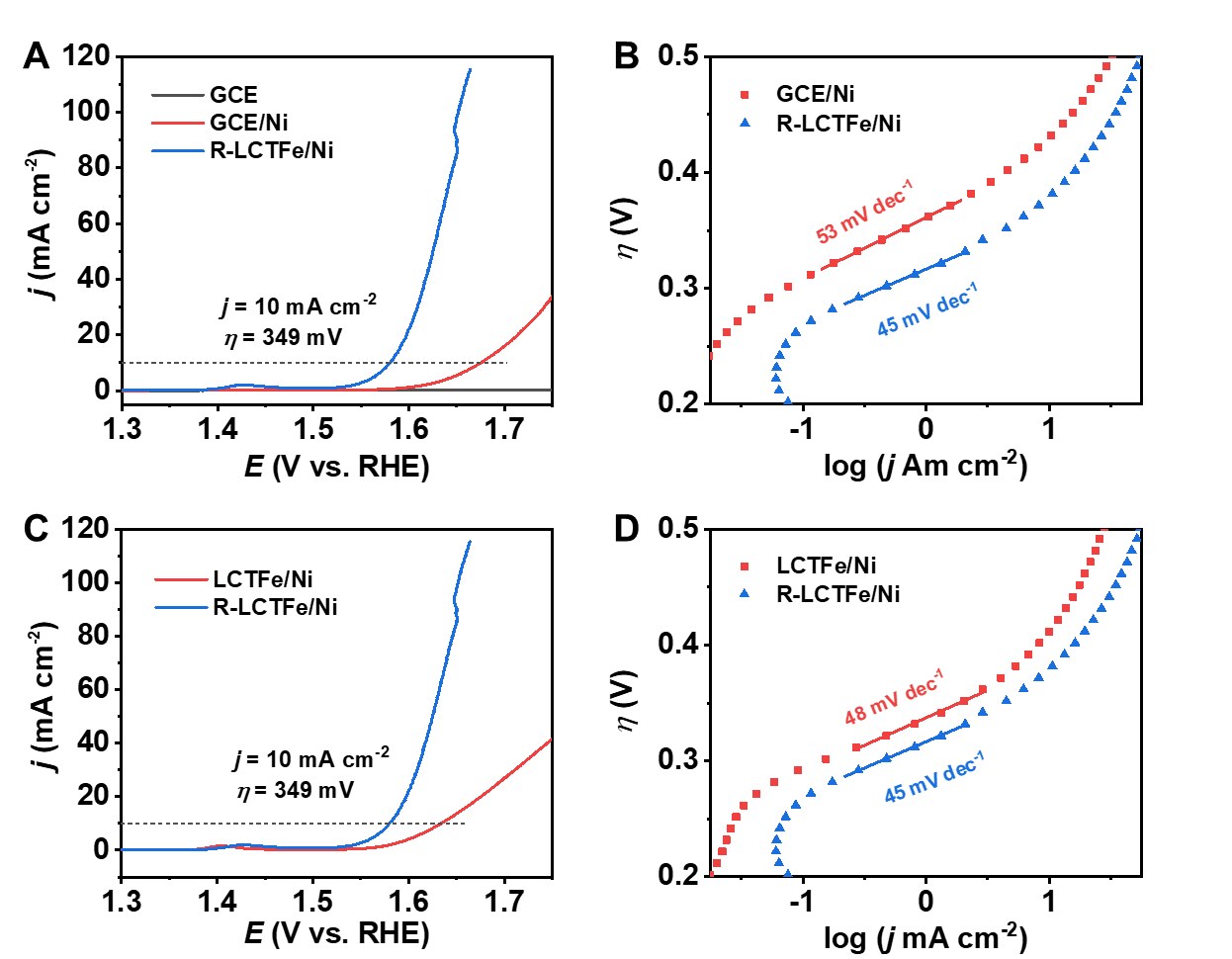


Figure S11. (A) OER LSV curves of GCE, GCE/Ni, and R-LCTFe/Ni in a 1.0 м KOH solution. (B) Corresponding Tafel plots (no GCE). (C) OER LSV curves and (D) corresponding Tafel plots of LCTFe/Ni and R-LCTFe/Ni in a 1.0 м KOH solution.


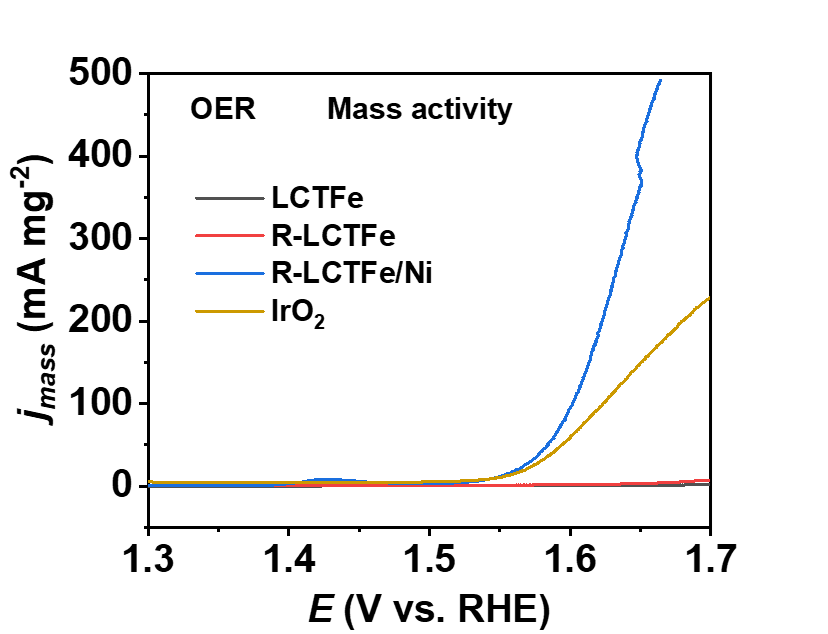


Figure S12. OER mass activity LSV curves of LCTFe, R-LCTFe, R-LCTFe/Ni and IrO_2_ in 1.0 м KOH solution.


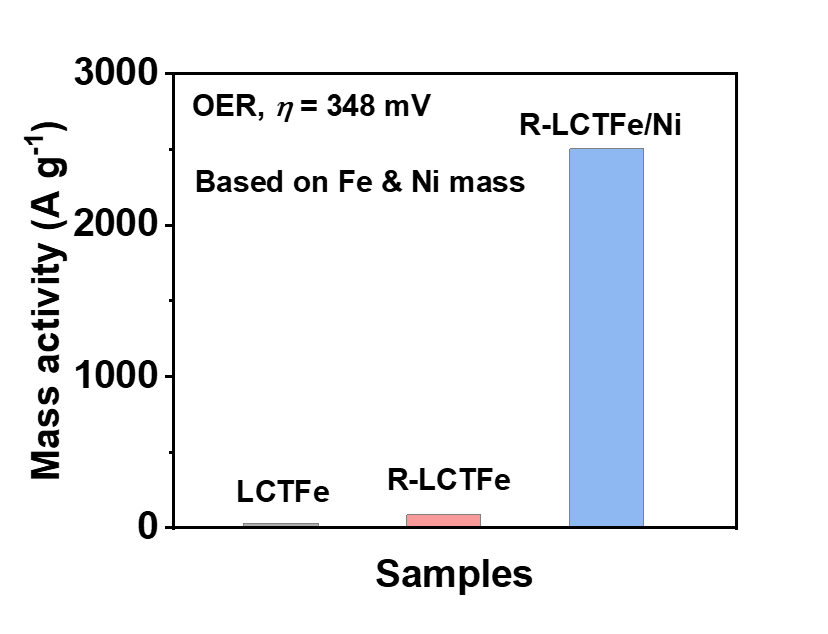


Figure S13. OER mass activities of LCTFe, R-LCTFe, and R-LCTFe/Ni based on Fe and Ni amount at an overpotential of 348 mV.

Figure S14. ECSA of LCTFe, R-LCTFe, and R-LCTFe/Ni.


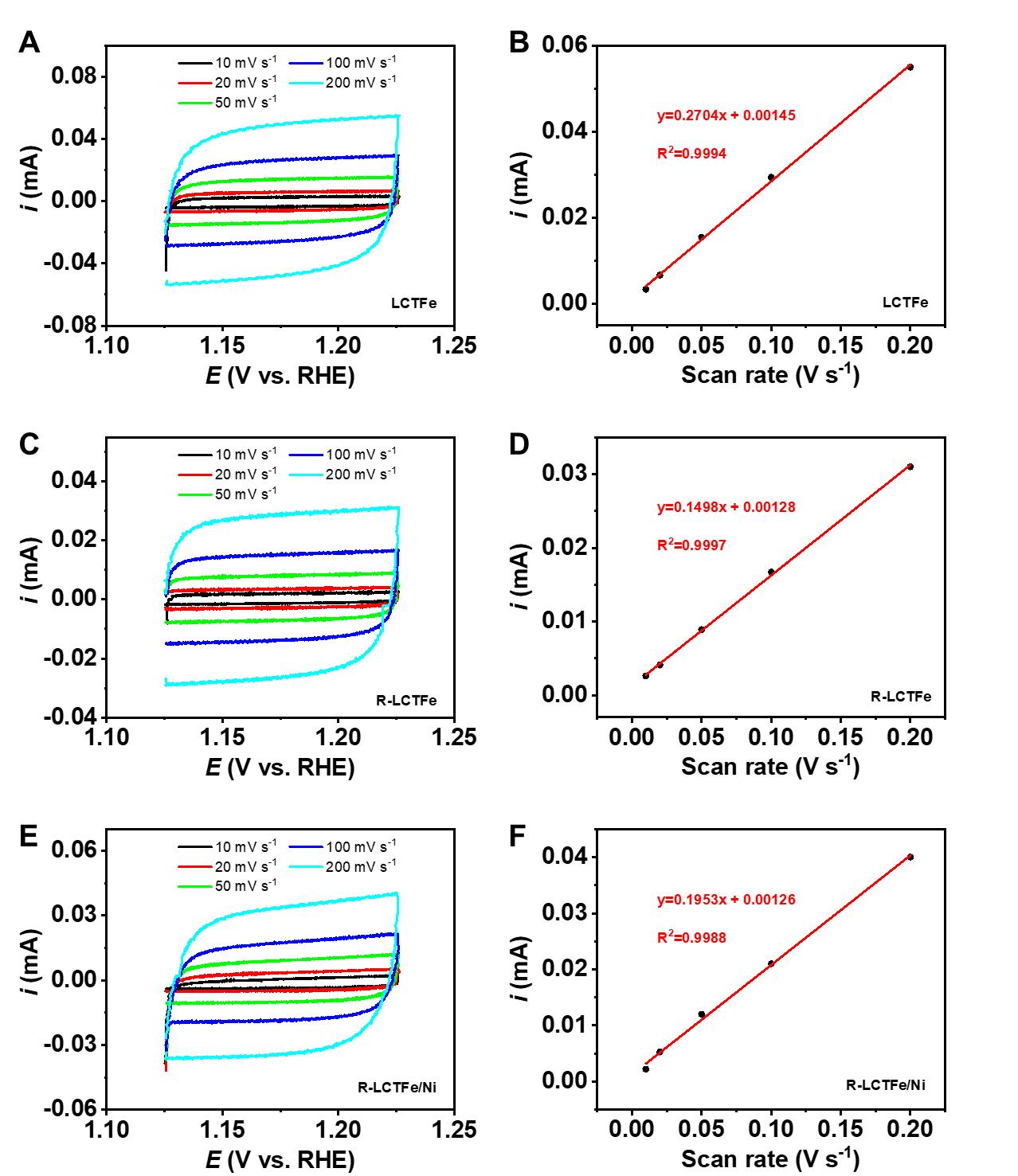


Figure S15. (A) Cyclic voltammograms of the LCTFe sample at scan rates ranging from 10 to 200 mV s^-1^ within the 1.13 to 1.23 V vs. RHE range. (B) Plot illustrating the correlation between scan rate and anodic current measured in the CV curves at 1.23 V vs. RHE. (C) CVs of the R-LCTFe sample at scan rates ranging from 10 to 200 mV s^-1^ within the 1.13 to 1.23 V vs. RHE range. (D) Plot illustrating the correlation between scan rate and anodic current measured in the CV curves at 1.23 V vs. RHE. (E) CVs of the R-LCTFe/Ni sample at scan rates ranging from 10 to 200 mV s^-1^ within the 1.13 to 1.23 V vs. RHE range. (F) Plot illustrating the correlation between scan rate and anodic current measured in the CV curves at 1.23 V vs. RHE.


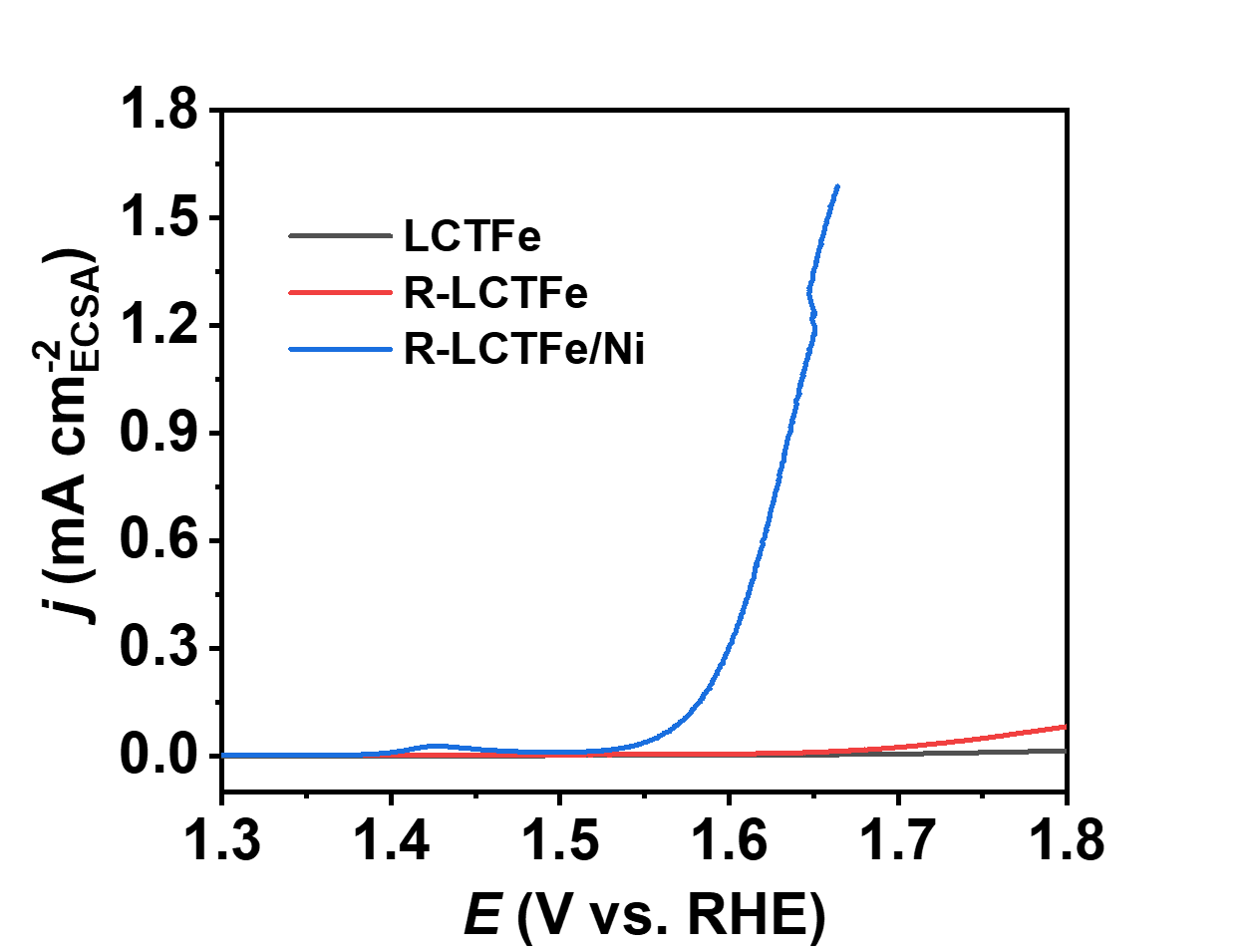


Figure S16. OER specific activity normalized to ECSA of LCTFe, R-LCTFe, and R-LCTFe/Ni.


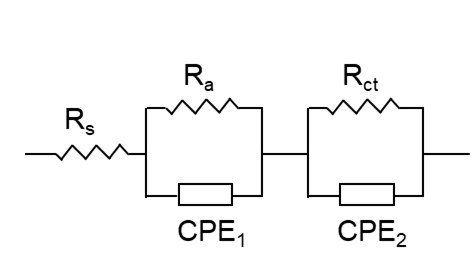


Figure S17. Equivalent Circuit (EC) used to fit EIS data.


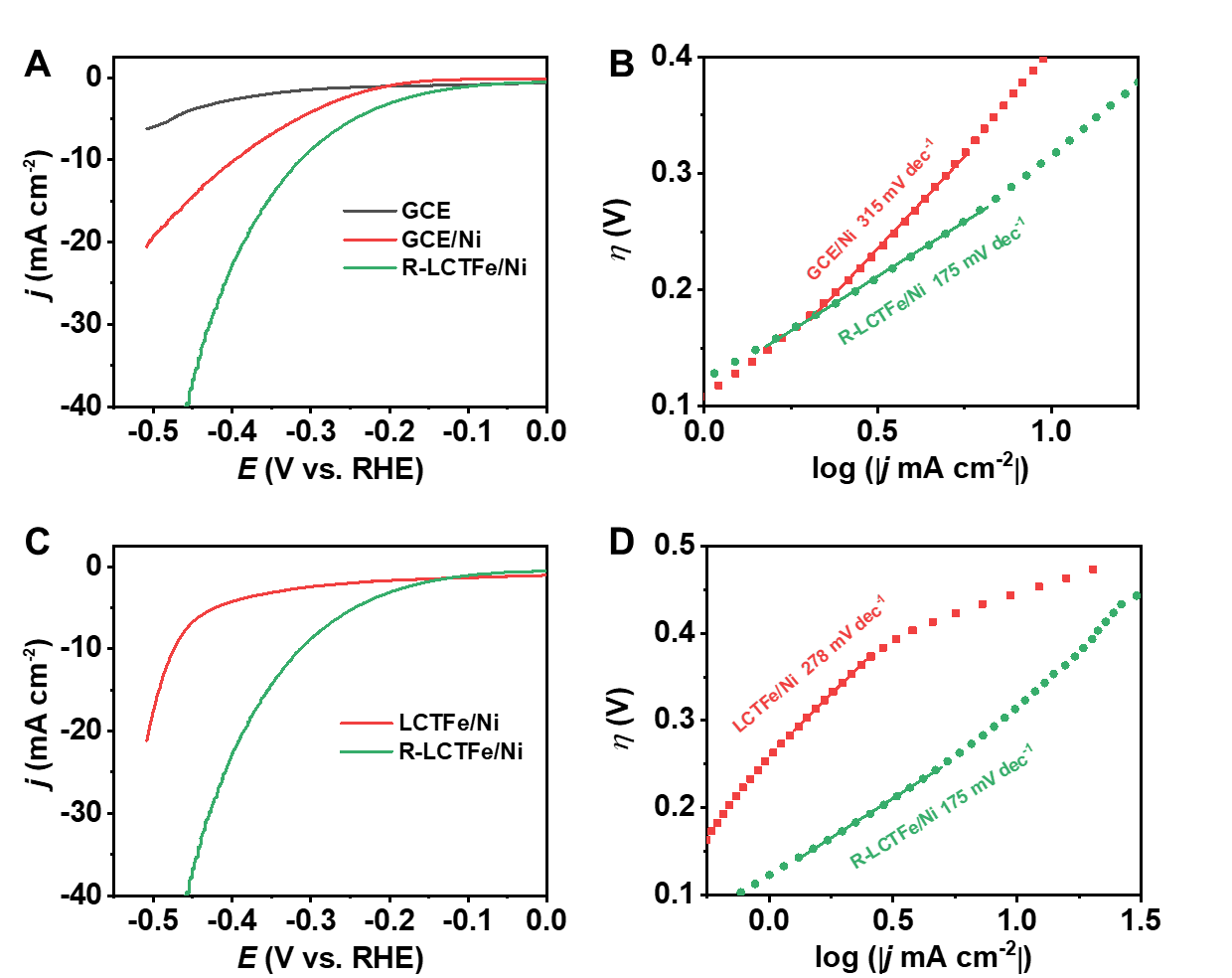


Figure S18. (A) HER LSV curves of GCE, GCE/Ni, and R-LCTFe/Ni in a 1.0 м KOH solution. (B) Corresponding Tafel plots (no GCE). (C) HER LSV curves and (D) corresponding Tafel plots of LCTFe/Ni and R-LCTFe/Ni in a 1.0 м KOH solution.


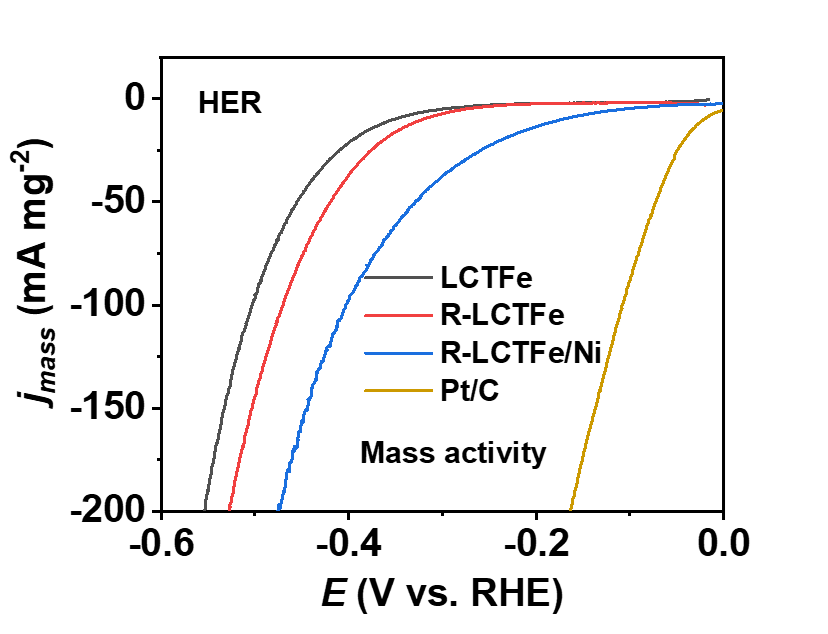


Figure S19. HER mass activity LSV curves of LCTFe, R-LCTFe, R-LCTFe/Ni and 20 wt% Pt/C in 1.0 м KOH solution.


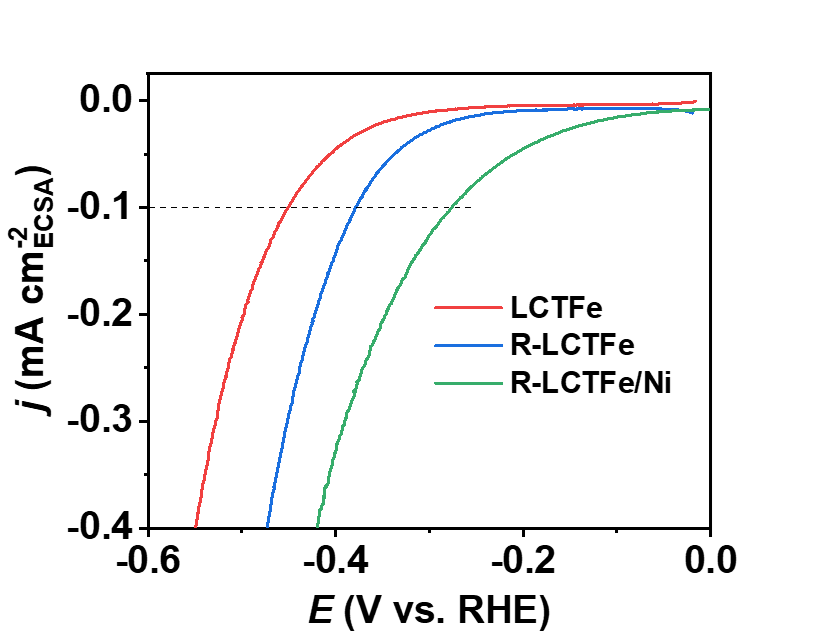


Figure S20. HER specific activity normalized to ECSA of LCTFe, R-LCTFe, and R-LCTFe/Ni.


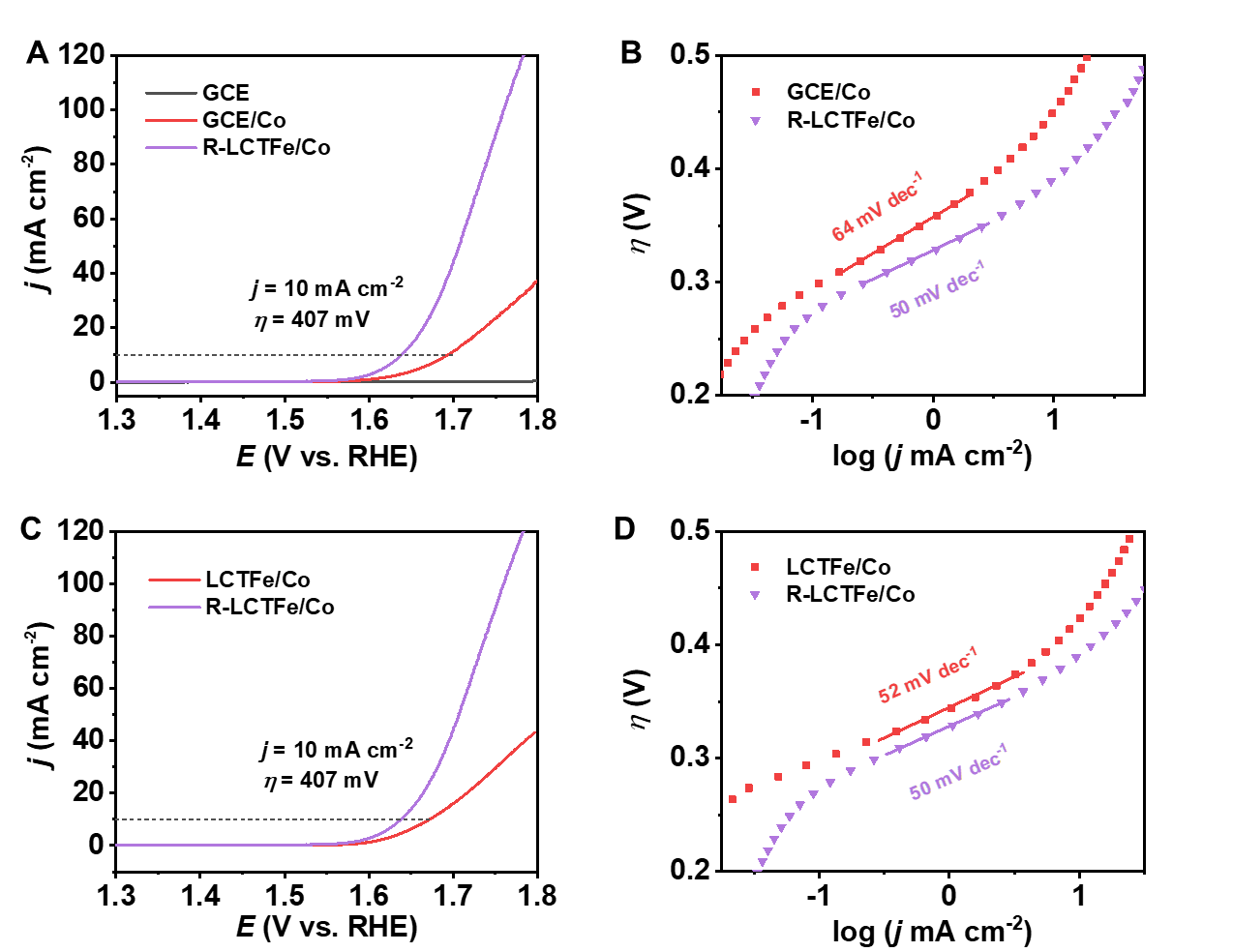


Figure S21. (A) OER LSV curves of GCE, GCE/Co, and R-LCTFe/Co in a 1.0 м KOH solution. (B) Corresponding Tafel plots (no GCE). (C) OER LSV curves and (D) corresponding Tafel plots of LCTFe/Co and R-LCTFe/Co in a 1.0 м KOH solution.


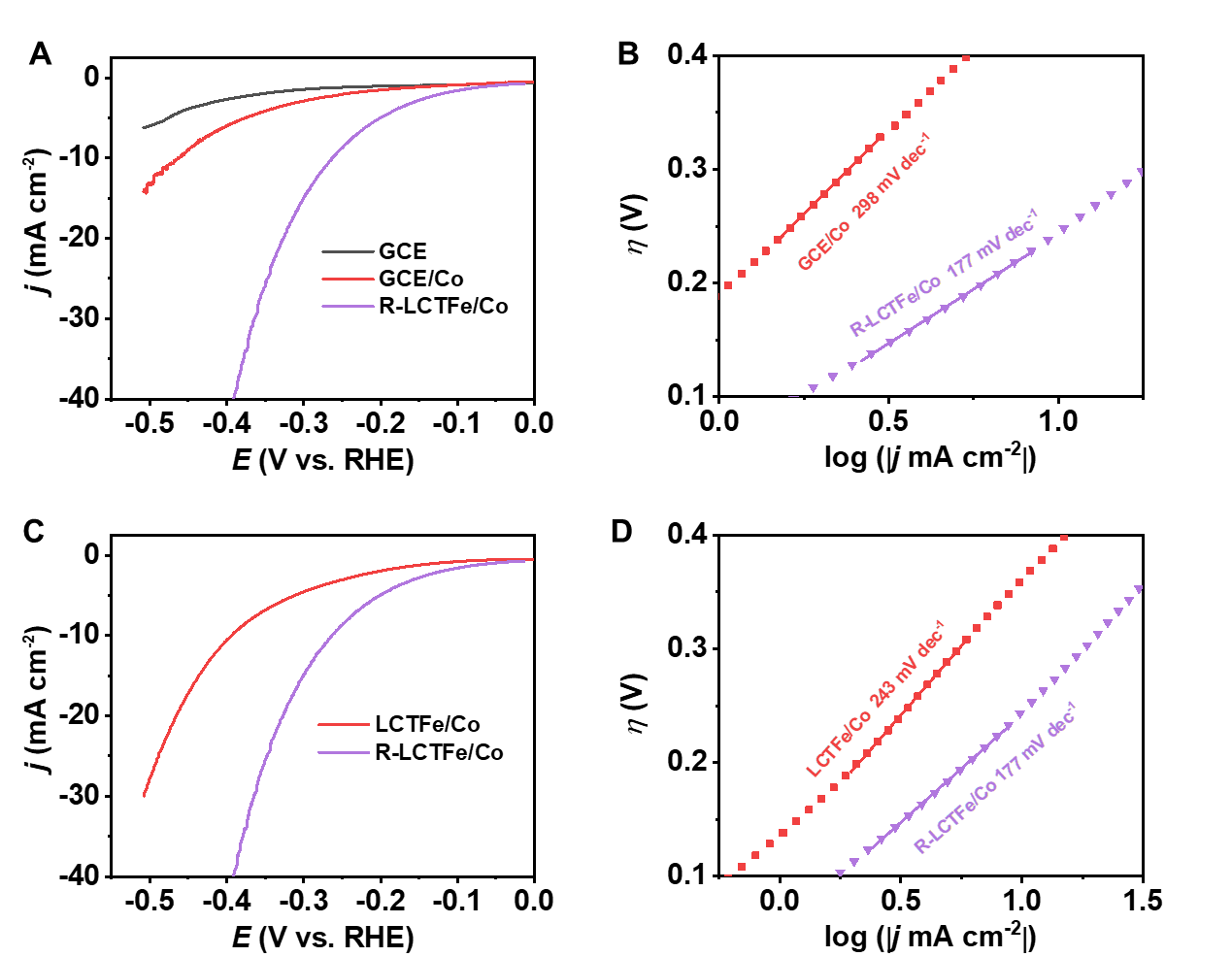


Figure S22. (A) HER LSV curves of GCE, GCE/Co, and R-LCTFe/Co in a 1.0 м KOH solution. (B) Corresponding Tafel plots (no GCE). (C) HER LSV curves and (D) corresponding Tafel plots of LCTFe/Co and R-LCTFe/Co in a 1.0 м KOH solution.

Table S1. OER activity comparison for R-LCTFe/Ni in this work with Perovskite-based catalysts in 1 м KOH

| **Catalyst** | **Overpotential** (mV)  at 10 mA cm^-2^ | **Tafel slope**  (mV dec^-1^) | **Mass activity** (A g^-1^)  at *η*_xxx(mV)_ | **Reference** |
| --- | --- | --- | --- | --- |
| **R-LCTFe/Ni** | **349** | **45** | **466 at *η*_418_** | **This work** |
| NBM_5.5_ | ca. 400 | 75 | 107 at *η*_470_ | ^[3]^ |
| LSCF-30 | 360 | 56.4 | ca.350 at *η*_420_ | ^[4]^ |
| LCC4 | 380 | 80 | - | ^[5]^ |
| 3DOM-LFC82 | 410 | 56 | 44 at *η*_410_ | ^[6]^ |
| La_0.9_Ce_0.1_NiO_3_ | 270 | 45 | - | ^[7]^ |
| A-PBCCF-H | 410 | 99 | - | ^[8]^ |
| LFNO-II NRs | 302 | 50 | - | ^[9]^ |
| La_0.7_Sr_0.3_CoO_3-δ_ | 326 | 70.8 | - | ^[10]^ |
| La_0.4_Sr_0.6_Ni_0.5_Fe_0.5_O_3_ | 320 | 52.77 | - | ^[11]^ |
| BSCF bulk | 420 | 104 | - | ^[12]^ |
| BSCF-NFs | 370 | 103 | - | ^[12]^ |
| CQDs@BSCF-NFs | 350 | 66 | - | ^[12]^ |

Table S2. The elemental ratio in LCTFe as determined from SEM EDS analysis.

| **Element** | **Line** | **Mass%** | **Atom%** |
| --- | --- | --- | --- |
| **La** | L | 31.12 ± 0.04 | 12.75 ± 0.14 |
| **Ca** | K | 25.13 ± 0.02 | 35.69 ± 0.24 |
| **Ti** | K | 41.23 ± 0.03 | 48.99 ± 0.29 |
| **Fe** | K | 2.52 ± 0.01 | 2.57 ± 0.08 |
| **Total** |  | 100.00 | 100.00 |

Table S3. The elemental ratio in R-LCTFe as determined from SEM EDS analysis.

| **Element** | **Line** | **Mass%** | **Atom%** |
| --- | --- | --- | --- |
| **La** | L | 31.33 ± 0.04 | 12.87 ± 0.14 |
| **Ca** | K | 24.67 ± 0.02 | 35.13 ± 0.24 |
| **Ti** | K | 41.46 ± 0.03 | 49.40 ± 0.30 |
| **Fe** | K | 2.54 ± 0.01 | 2.59 ± 0.08 |
| **Total** |  | 100.00 | 100.00 |

Table S4. Values of components in EC of LCTFe, R-LCTFe, and R-LCTFe/Ni in OER.

| **Sample** | **Element** | **Value** | **Error %** |
| --- | --- | --- | --- |
| **LCTFe** | R_s_ (Ω) | 16.71 | 1.06 |
|  | R_a_ (Ω) | 47.76 | 7.70 |
|  | CPE_1_-T | 0.0015 | 12.14 |
|  | CPE_1_-P | 0.44 | 3.12 |
|  | **Rct (Ω)** | **3205** | 0.78 |
|  | CPE_2_-T | 1.6E-4 | 0.68 |
|  | CPE_2_-P | 0.93 | 0.35 |
| **R-LCTFe** | R_s_ (Ω) | 10.67 | 0.91 |
|  | R_a_ (Ω) | 107.2 | N/A |
|  | CPE_1_-T | 1.6E-3 | 10.51 |
|  | CPE_1_-P | 0.50 | 2.27 |
|  | **Rct (Ω)** | **609.1** | 1.52 |
|  | CPE_2_-T | 2.9E-4 | 2.19 |
|  | CPE_2_-P | 0.85 | 1.35 |
| **R-LCTFe/Ni** | R_s_ (Ω) | 16.69 | 1.35 |
|  | R_a_ (Ω) | 7.03 | 2.55 |
|  | CPE_1_-T | 4.3E-4 | 2.41 |
|  | CPE_1_-P | 0.93 | 8.1 |
|  | **Rct (Ω)** | **10.73** | 1.49 |
|  | CPE_2_-T | 5.5E-3 | 4.15 |
|  | CPE_2_-P | 0.42 | 1.21 |

Table S5. Values of components in EC of LCTFe, R-LCTFe, and R-LCTFe/Ni in HER.

| **voltage** | **Element** | **Value** | **Error %** |
| --- | --- | --- | --- |
| **LCTFe** | R_s_ (Ω) | 10.09 | 0.52 |
|  | R_a_ (Ω) | 79.24 | 9.47 |
|  | CPE_1_-T | 0.001 | 5.25 |
|  | CPE_1_-P | 0.63 | 1.27 |
|  | **Rct (Ω)** | **651** | 3.05 |
|  | CPE_2_-T | 0.001 | 1.94 |
|  | CPE_2_-P | 1.004 | 1.77 |
| **R-LCTFe** | R_s_ (Ω) | 17.84 | 0.37 |
|  | R_a_ (Ω) | 50.64 | 3.90 |
|  | CPE_1_-T | 5.9E-4 | 5.08 |
|  | CPE_1_-P | 0.76 | 1.07 |
|  | **Rct (Ω)** | **575.7** | 1.22 |
|  | CPE_2_-T | 7.6E-4 | 1.02 |
|  | CPE_2_-P | 0.98 | 0.75 |
| **R-LCTFe/Ni** | R_s_ (Ω) | 12.76 | 0.33 |
|  | R_a_ (Ω) | 90.17 | 7.95 |
|  | CPE_1_-T | 0.003 | 7.61 |
|  | CPE_1_-P | 0.91 | 4.35 |
|  | **Rct (Ω)** | **164.1** | 3.42 |
|  | CPE_2_-T | 3.2E-4 | 2.05 |
|  | CPE_2_-P | 0.71 | 0.44 |

Reference

[1] T. Zhang, Q. Ye, Z. Han, Q. Liu, Y. Liu, D. Wu, H. J. Fan, *Nat. Commun.*, **2024**, *15*, 6508.

[2] X. Bo, R. K. Hocking, S. Zhou, Y. Li, X. Chen, J. Zhuang, Y. Du, C. Zhao, *Energy Environ. Sci.*, **2020**, *13*, 4225-4237.

[3] J. Wang, Y. Gao, D. Chen, J. Liu, Z. Zhang, Z. Shao, F. Ciucci, *ACS Catal.*, **2018**, *8*, 364-371.

[4] R. Zong, Y. Fang, C. Zhu, X. Zhang, L. Wu, X. Hou, Y. Tao, J. Shao, *ACS Appl. Mater. Interfaces*, **2021**, *13*, 42852-42860.

[5] D. Ji, C. Liu, Y. Yao, L. Luo, W. Wang, Z. Chen, *Nanoscale*, **2021**, *13*, 9952-9959.

[6] J. Dai, Y. Zhu, Y. Zhong, J. Miao, B. Lin, W. Zhou, Z. Shao, *Adv. Mater. Interfaces*, **2019**, *6*, 1801317.

[7] Y. Sun, R. Li, X. Chen, J. Wu, Y. Xie, X. Wang, K. Ma, L. Wang, Z. Zhang, Q. Liao, *Adv. Energy Mater.*, **2021**, *11*, 2003755.

[8] B. Hua, M. Li, Y.-F. Sun, Y.-Q. Zhang, N. Yan, J. Chen, T. Thundat, J. Li, J.-L. Luo, *Nano Energy*, **2017**, *32*, 247-254.

[9] H. Wang, J. Wang, Y. Pi, Q. Shao, Y. Tan, X. Huang, *Angew. Chem.*, **2019**, *131*, 2338-2342.

[10] Y. Lu, A. Ma, Y. Yu, R. Tan, C. Liu, P. Zhang, D. Liu, J. Gui, *ACS Sustain. Chem. Eng.*, **2018**, *7*, 2906-2910.

[11] Q. Guo, X. Li, H. Wei, Y. Liu, L. Li, X. Yang, X. Zhang, H. Liu, Z. Lu, *Front. Chem.*, **2019**, *7*, 224.

[12] G. Li, S. Hou, L. Gui, F. Feng, D. Zhang, B. He, L. Zhao, *Appl. Catal. B-Environ.*, **2019**, *257*, 117919.
